# Triphenylphosphonium conjugated gold nanotriangles impact Pi3K/AKT pathway in breast cancer cells: a photodynamic therapy approach

**DOI:** 10.1038/s41598-023-28678-x

**Published:** 2023-02-08

**Authors:** Nadar Manimaran Vinita, Umapathy Devan, Sabapathi Durgadevi, Selvaraj Anitha, Dhamodharan Prabhu, Sundarraj Rajamanikandan, Muthusamy Govarthanan, Ananthanarayanan Yuvaraj, Muniyandi Biruntha, Arockiam Antony Joseph Velanganni, Jeyaraman Jeyakanthan, Pitchan Arul Prakash, Mohamed Sultan Mohamed Jaabir, Ponnuchamy Kumar

**Affiliations:** 1 Food Chemistry and Molecular Cancer Biology Lab, Department of Animal Health and Management, Alagappa University, Karaikudi, Tamil Nadu 630 003 India; 2 Molecular Oncology Laboratory, Department of Biochemistry, Bharathidasan University, Tiruchirappalli, Tamil Nadu 620 024 India; 3 Research and Development Wing, Sree Balaji Medical College and Hospital (SBMCH), Bharath Institute of Higher Education and Research (BIHER), Chrompet, Chennai, Tamil Nadu 600 044 India; 4 Department of Biochemistry, Centre for Drug Design, Karpagam Academy of Higher Education, Coimbatore, Tamil Nadu 641 021 India; 5 Department of Environmental Engineering, Kyungpook National University, Deagu, 41566 Republic of Korea; 6 Department of Biomaterials, Saveetha Dental College and Hospital, Saveetha Institute of Medical and Technical Sciences, Chennai, Tamil Nadu 600 077 India; 7 Department of Zoology, Periyar University, Salem, Tamil Nadu 636 011 India; 8 Department of Bioinformatics, Alagappa University, Karaikudi, Tamil Nadu 630 003 India; 9 PG and Research Department of Biotechnology and Microbiology, The National College, Tiruchirappalli, Tamil Nadu 620 001 India

**Keywords:** Materials science, Biomaterials

## Abstract

Although gold nanoparticles based photodynamic therapy (PDT) were reported to improve efficacy and specificity, the impact of surface charge in targeting cancer is still a challenge. Herein, we report gold nanotriangles (AuNTs) tuned with anionic and cationic surface charge conjugating triphenylphosphonium (TPP) targeting breast cancer cells with 5-aminoleuvinic acid (5-ALA) based PDT, in vitro. Optimized surface charge of AuNTs with and without TPP kill breast cancer cells. By combining, 5-ALA and PDT, the surface charge augmented AuNTs deliver improved cellular toxicity as revealed by MTT, fluorescent probes and flow cytometry. Further, the 5-ALA and PDT treatment in the presence of AuNTs impairs cell survival Pi3K/AKT signaling pathway causing mitochondrial dependent apoptosis. The cumulative findings demonstrate that, cationic AuNTs with TPP excel selective targeting of breast cancer cells in the presence of 5-ALA and PDT.

## Introduction

Despite significant lab and clinical research, the prevalence of breast cancer continues to rise, inflicting misery on women^1,2^. As a result, low- and middle-income countries face several obstacles in cancer detection, diagnosis, and their related therapies^3–5^. Hence, providing patients with effective drugs that minimize harmful effects is the need of the hour. To combat this issue, nanotechnology has emerged as a superior platform for the simultaneous delivery of drugs^6–9^.

In this journey, therapeutic nanoparticles selectively target tumors, enhance anticancer effectiveness and circumventing drug resistance^10–12^. For instance, gold nanoparticles are among the most promising agents for cancer treatment due to these reasons; (i) small and capable of selectively penetrating cancer cells through the enhanced permeability and retention (EPR) effect^13,14^ (ii) their ability to bind proteins and drugs in targeting cancer cells that possess cell surface receptors^15,16^ (iii) can absorb light radiations and provide better contrast images than conventional agents^17,18^.

Over the last few decades, gold nanoparticles have been conjugated with various functionalizing moieties, including ligands, therapeutic agents, DNA, amino acids, proteins, peptides, oligonucleotides, etc^19–22^. Recently, a research study demonstrated the utilization of PEG ligands with gold nanoparticles to functionalize doxorubicin (DOX) at low pH^23^. Similarly, phthalocyanines were functionalized on the surface of gold nanoparticles for efficient oxygen production^24^.

A few research has also shown that gold nanoparticles of varying shapes may display compelling surface plasmon resonance when exposed to light^25,26^. Considering the fact, photothermal ablation using gold nanoparticles has been tested on various target cells, including cancer^27–29^. Moreover, light-induced heating may be utilized to release therapeutic drugs coupled with gold nanoparticles^30^. Altogether, it has been shown that gold nanoparticles may be employed successfully in photodynamic therapy (PDT), in which light is used to promote localized singlet oxygen production^31,32^.

In view of the above, the most popular choice for PDT with gold nanoparticles is 5-aminolaevulinic acid (5-ALA), a naturally occurring amino acid. Recent studies have shown that PDT using nanoparticles coated with 5-ALA causes minimal harm to fibroblasts and kills cancer cells effectively^33^. In addition, PDT of K562 cells with 5-ALA linked gold nanoparticles was also shown to be efficient^34^. Likewise, PDT involving 5-ALA with gold nanoparticles results in a significant decrease in cancer cells^34^.

In the meanwhile, targeting essential cellular organelles can boost the therapeutic index of PDT by enhancing its efficacy^35–38^. To achieve this, mitochondria are considered a potential therapeutic target for several diseases, including cancer^39,40^. This is possible by conjugating exogenous molecules to the surface of gold nanoparticles. For example, utilizing lipophilic cationic molecules (such as triphenylphosphonium, TPP) may act selectively on mitochondria can be considered for cancer therapy^41^. A recent study has shown that nanoparticles functionalized with TPP target CHO and HeLa cells for sub-cellular imaging^42^. Similarly, increased PDT has been accomplished by targeting breast cancer cells with gold nanoparticles incorporating TPP^43^.

In light of the above, the present study attempt to synthesize gold nanotriangles (AuNTs) with positive and negative surface charges followed by TPP functionalization for PDT in the presence of 5-ALA. In addition, the molecular mechanism that drives the demise of breast cancer cells was also explored. To our knowledge, this is the first-time mitochondria have been targeted precisely using AuNTs@TPP with enhanced effectiveness of PDT.

## Materials and methods

### Chemicals

Sigma-Aldrich, India provided Chloroauric acid (HAuCl_4_.3H_2_O), cetyltrimethylammonium chloride (CTAC), sodium poly(styrene sulfonate) (PSS, Mw: 70 kDa) and methyltriphenylphosphonium bromide. HiMedia Laboratories India provided cell cultured based media and chemicals. Primers and antibodies used in the study were received from Eurofins and Cell-signaling technologies. India, respectively.

### Instruments

Optical studies were conducted in UV–*vis* spectrophotometer (Evolution, 201, Thermo, USA), Fourier transform infrared (FTIR, Nicholet is5, Thermo, USA), Fluorescence spectroscopy (HORIBA Fluromax 4, USA), Micro-Raman spectroscopy (Seiki, Japan) and Inductively coupled plasma emission spectroscopy (ICPE-9800 series). Diffraction pattern based studies were carried out in X-ray diffraction (X’Pert Pro-PAnalytic, UK), X-ray photoelectron (XPS, PHI-VERSAPROBE III, USA) and Energy dispersive X-ray spectroscopy (EDS, TESCAN OXFORD). Surface morphology of nanomaterials were ascertained by High-resolution-transmission electron microscope (HR-TEM, Joel-2100) attached with selected area energy diffraction (SAED). The hydrodynamic diameter and zeta potential was carried out by Zetasizer, Nano-Zs90, Malvern, UK.

### Synthesis of gold nanotriangles (AuNTs)

In the present study, cationic AuNTs (CTAC©AuNTs) were prepared according to the method outlined by Bhattarai et al.^44^. Further, the surface charge was modified by allowing anionic PSS (2 mg/mL in 6 mM NaCl) to react with CTAC©AuNTs (50 µg/mL), thereafter centrifuging at 12,000 rpm for 15 min to remove excess unbound PSS. The obtained CTAC©AuNTs and PSS@CTAC©AuNTs were stored at 4 °C until before use. The concentration of AuNTs prepared in this study was determined using ICP-OES analysis.

### Development of gold nanoconjugates

To facilitate conjugation, two types of surface charge-impregnated gold nanotriangles (CTAC©AuNTs and PSS@CTAC©AuNTs) were allowed to react with TPP (0–300 µg/mL) for 3 h^45^. After the completion of the reaction, the unbound TPP was separated using centrifugation at 12,000 rpm for 15 min. The final product thus obtained was redispersed in 2 mL of sterile distilled water and stored (4 °C) for further use. In addition, high-throughput characterization of the conjugated entities was performed to evaluate their loading efficiency and surface charge. In our study, TPP means thiolated-TPP which was synthesized following the methodology of Yang et al.^43^.

### Cell culture

The cells used in the study were purchased from National Centre for Cell Science (NCCS), Pune, India. The obtained cells were cultured in Dulbeco's modified eagle's medium (DMEM) with 10% fetal bovine serum (FBS) and 1% antibiotics (Streptomycin/Penicillin) under a humidified environment (37 °C with 5% CO_2_) for various assays and then harvested using trypsinization.

### Cell cytotoxicity assay

In the present study, the cytotoxicity of gold nanoconjugates (CTAC©AuNTs and PSS@CTAC©AuNTs) for 24 h were assessed by MTT assay against breast cancer cells (MCF-7 and MDA-MB-231)^46^.

### PDT treatment

For PDT treatment, the normal and cancer cells were incubated with photo-sensitizer, 5-ALA (0.5 mM) for 4 h in serum free media. After incubation, the media containing 5-ALA was replaced with fresh serum free media and nanoconjugates (TPP-CTAC©AuNTs and TPP-PSS@CTAC©AuNTs). Subsequently, the cells with nanoconjugates were irradiated using a halogen lamp for 1 min and incubated for 24 h. After 24 h, MTT assay was performed to study the effect of PDT^46^. Similar experiments were carried without irradiation to authenticate PDT efficiency.

### Cytotoxicity assay

The cytotoxicity of gold nanoconjugates on breast cancer cells (MCF-7 and MDA-MB-231) was evaluated by MTT assay for 24 h in a dose-dependent manner. In addition, the percentage of cell viability was calculated by measuring absorbance at 595 nm using a microtiter plate reader (BIORAD, USA). In addition, HEK-293 cells were used to assess the biocompatibility of gold nanoconjugates.

### Detection of apoptosis utilizing fluorescent probes

To assess the morphological alterations in breast cancer cells (MCF-7 and MDA-MB-231) during the event of apoptosis, fluorescent probes such as Acridine Orange/Ethidium bromide (AO/EtBr, dual), Hoechst 33342 (nuclear), propidium iodide (PI), Rhodamine 123 (mitochondrial membrane potential, ΔΨm) and 2′,7′-Dichlorofluorescein diacetate (DCFH-DA, Reactive Oxygen Species) staining was performed upon treatment with various gold nanoconjugates. Fluorescence microscopic studies were recorded using an Accu-Scope EXI-310 microscope.

### PpIX formation

In the present study, the intracellular PpIX was measured in breast cancer cells (MCF-7 and MDA-MB-231) using a serum free medium containing gold nanoconjugates and 0.5 mM 5-ALA, followed by irradiation. Cultures without treatment were used as control. The PpIX kinetics was measured using a microtiter plate reader at different time intervals based on the methodology reported elsewhere^43^.

### Cell cycle analysis

The cell cycle pattern alteration in breast cancer cells (MCF-7 and MDA-MB-231) was studied by using a flow cytometer in the presence of PI. After 24 h, PDT-treated cells were fixed (4 mL ice-cold ethanol), stained (0.5 mL of PI), and subjected to flow cytometric measurements.

### Annexin-V FITC/PI apoptosis assay

After PDT, the progression of apoptosis in breast cancer cells (MCF-7 and MDA-MB-231) was ascertained by employing the Annexin V-FITC kit. First, the breast cancer cells were harvested and washed with PBS (thrice), and about 5 µL of Annexin-V-FITC reagent was added, followed by incubation for 10 min dark. Later, the cells were washed with PBS and resuspended in binding buffer (190 µL) and propidium iodide (10 µL) solution. Finally, the obtained mixture was instantly subjected to flow cytometer analysis followed by data processing.

### Co-localization experiment

Micro-raman analysis was performed to ascertain the co-localization of TPP within the breast cancer cells^43^. Briefly, the cells treated with gold nanoconjugates in petri dishes were subjected to Raman spectroscopy in PBS between 300 and 4500 cm^−1^. All spectra are baseline corrected for PBS solution and petri dish glass bottom.

### Gene expression studies

After PDT, the mRNA from breast cancer cells (MCF-7 and MDA-MB-231) was extracted using a Trizol reagent^47^. cDNA was constructed using PrimeScript 1st strand cDNA Synthesis kit one-step RT-PCR (Takara, Japan). Next, the cDNA was amplified using genes for which the details of the primers were listed in Supporting Information SI, Table. 1. The final product was subjected to electrophoresis, and gels were visualized using GE Image Quant LAS 500, USA, followed by quantification of band intensity by ImageJ Software.

### Western blot analysis

Following PDT therapy, breast cancer cells (MCF-7 and MDA-MB-231) treated with gold nanoconjugates were subjected to western blot analysis to examine their protein levels^47^. Proteins were extracted, quantified, separated (on a 12% gel), and transferred to a nitrocellulose membrane (50 mV for 1 h).The nitrocellulose membrane was then blocked (5% BSA in TBA for 2 h), incubated with the primary antibody for 12 h, and washed three times in tris-buffered saline with 0.2% Tween-20 (TBST, every 10 min). After washing, the nitrocellulose membrane was further incubated with a secondary antibody (for 1 h) before being washed (three times) with TBST. Chromogenic chemicals were used to develop the blots, and a GE Image Quant LAS 500, USA, was used for visualization. In addition, ImageJ software was used to analyze the band's intensity quantitatively.

### Statistical analysis

All experiments carried out in our study were repeated at least three times (Mean ± SE). Using two-way ANOVA, a p-value of experiments was carried out at a 5% confidence level using GraphPad Prism 8 software.

## Result and discussion

### Synthesis of positively charged gold nanotriangles (CTAC©AuNTs)

**Figure 1:**
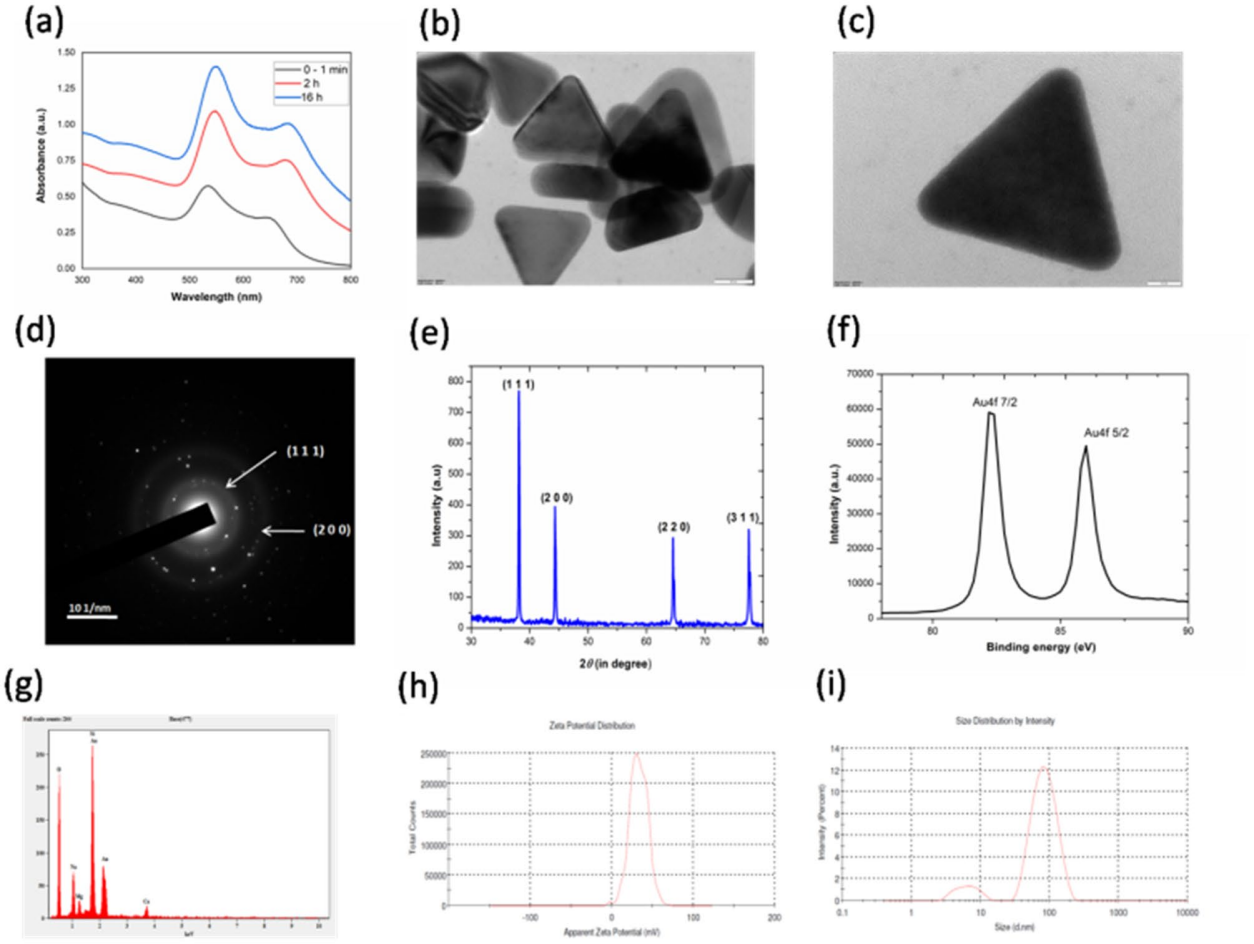
Optical and structural characterization of CTAC©AuNTs in DI water. (**a**) UV–*vis* absorption spectra at three different reaction times (0–1 min, 2 h, and 16 h). (**b**, **c**) TEM and HR-TEM micrographs. (**d**) SAED pattern. (**e**) X-ray diffraction pattern (JCPDS No. 04-0784). (**f**) A typical XPS spectrum. (**g**) Elemental mapping by EDAX. (**h**) Apparent zeta potential value (+ 33 ± 2.9 mV). (**i**) Size distribution by intensity (50.33 ± 6.69 nm).

In the present study, positively charged AuNTs were produced by a seed-mediated method based on the Murphy preparation of AuNTs, which includes an intermediate and final growth solution^44^. The following are the outcomes of blending two reaction mixtures: Fig. 1a illustrates the UV–*vis* absorption spectra of the initial seed solution prior to the synthesis of CTAC©AuNTs (0–1 min). When the growth solution is added, a distinct surface plasmon resonance (SPR) at 548 nm designates the growth of CTAC©AuNTs between 2 and 16 h, as shown in Fig. 1a. High resolution—transmission electron micrograph (HR-TEM) shows the synthesis of highly pristine CTAC©AuNTs with sizes varying from 20 to 25 nm (Fig. 1b and c). The selected area electron diffraction (SAED) pattern shows two concentric face-centred cubic (FCC) rings that correspond to the crystal planes for gold at (111) and (200) (Fig. 1d). At the same time, the XRD diffraction peaks of CTAC©AuNTs exhibit FCC crystalline phase with JCPDS No. 04-0784 (Fig. 1e). Figure 1f represents high-resolution XPS spectra which disclose the presence of doublet peaks for gold with binding energies of 82.28 and 85.99 eV, corresponding to Au4f 7/2 and Au4f 5/2, respectively. Energy-dispersive X-ray (EDX) analysis (Fig. 1g) also confirms the existence of signals from gold. The capping of CTAC on the surface of AuNTs provided a positive surface charge of + 33 ± 2.9 mV (Fig. 1h). The hydrodynamic diameter of CTAC©AuNTs was 50.33 ± 6.69 nm (Fig. 1i). These findings were consistent with previous studies on the nucleation of CTAC©AuNTs^44,48^.

### Synthesis of negatively charged gold nanotriangles (PSS@CTAC©AuNTs)

**Figure 2:**
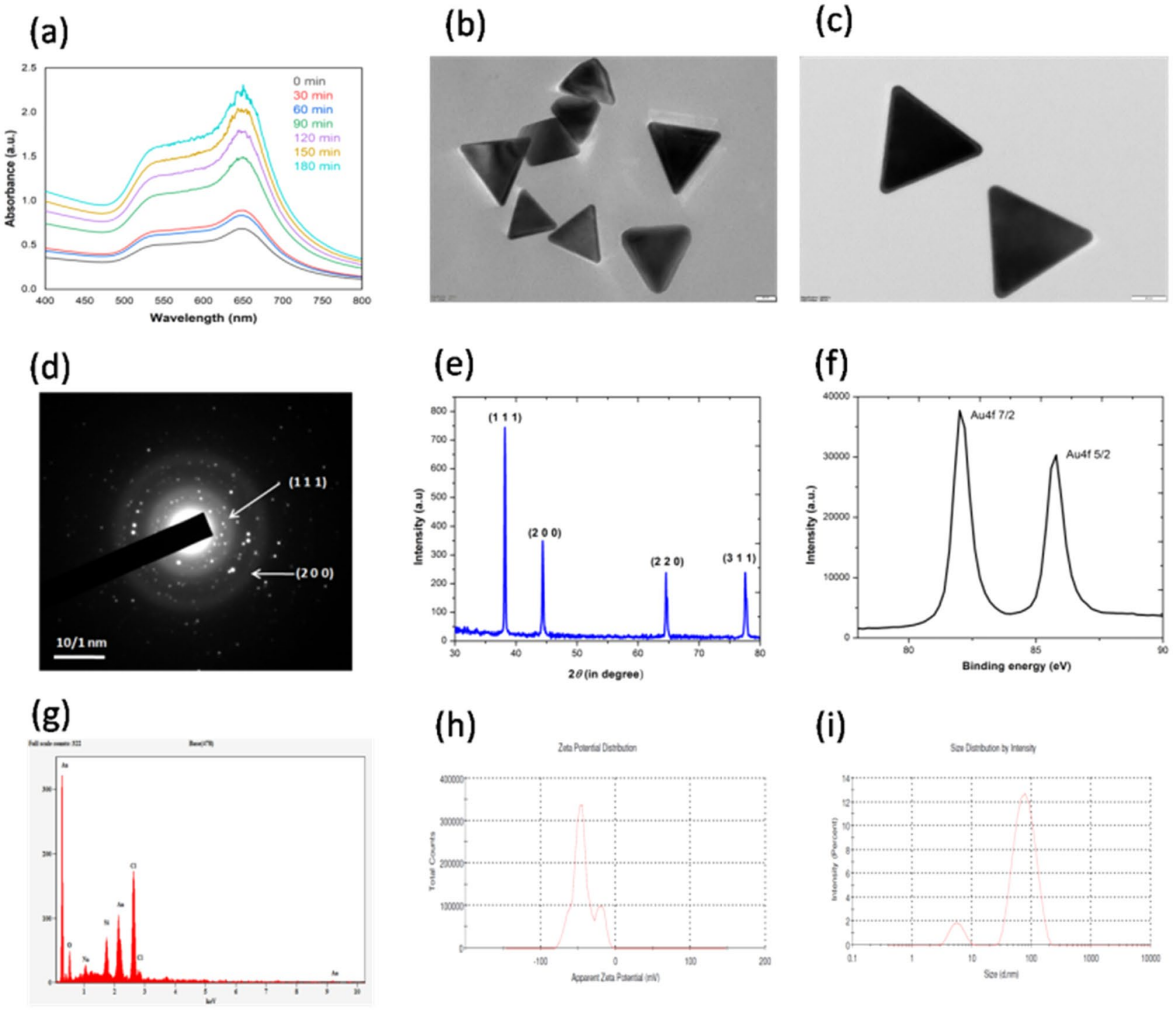
Optical and structural characterization of PSS@CTAC©AuNTs in DI water. (**a**) UV–*vis* absorption spectra at various time intervals (0–180 min). (**b**, **c**) TEM and HR-TEM micrographs. (**d**) SAED pattern. (**e**) X-ray diffraction pattern (JCPDS No. 04-0784). (**f**) A typical XPS spectrum. (g) Elemental mapping by EDAX. (h) Apparent zeta potential value (− 42.9 ± 5.45 mV). (**i**) Size distribution by intensity (51.643 ± 8.8 nm).

In this section, an anionic surfactant polystyrene sulfonate (PSS) was coated onto the surface of CTAC©AuNTs to yield negatively charged AuNTs (PSS@CTAC©AuNTs)^45^. UV–*vis* absorption spectral analysis confirms the conjugation of PSS onto the surface of CTAC©AuNTs with a redshift in surface plasmon resonance (650 nm) (Fig. 2a). HR-TEM analysis of PSS@CTAC©AuNTs revealed a similar structure to CTAC©AuNTs with an increased size (25–30 nm) due to coating with PSS (Fig. 2b and c). SAED pattern confirmed the crystallinity of PSS@CTAC©AuNTs with reference to FCC gold rings at (111), (200), (220), and (311) (Fig. 2d). The XRD measurement of PSS@CTAC©AuNTs (Fig. 2e) with JCPDS No. 04-0784 is well in agreement with SAED studies. XPS analysis surmounts a shift in binding energy for PSS@CTAC©AuNTs at 82.25 and 85.85 eV for gold (Fig. 2f). The EDX measurement of PSS@CTAC©AuNTs validates the occurrence of metallic gold (Fig. 2g). Undeniably, the coating of PSS on the surface of CTAC©AuNTs provides a negative surface charge value of − 42.9 ± 9.88 mV (Fig. 2h) with an average hydrodynamic diameter of 51.64 ± 5.79 nm (Fig. 2i). Meanwhile, the polyelectrolyte coating of PSS provides better stability and biocompatibility for the AuNTs^49^. However, the toxicology aspect of CTAC©AuNTs and PSS@CTAC©AuNTs needs to be addressed. The results from the study are consistent with earlier reports on the preparation of gold nanorods using a PSS^45^.

### Conjugation of TPP on the surface of AuNTs

**Figure 3:**
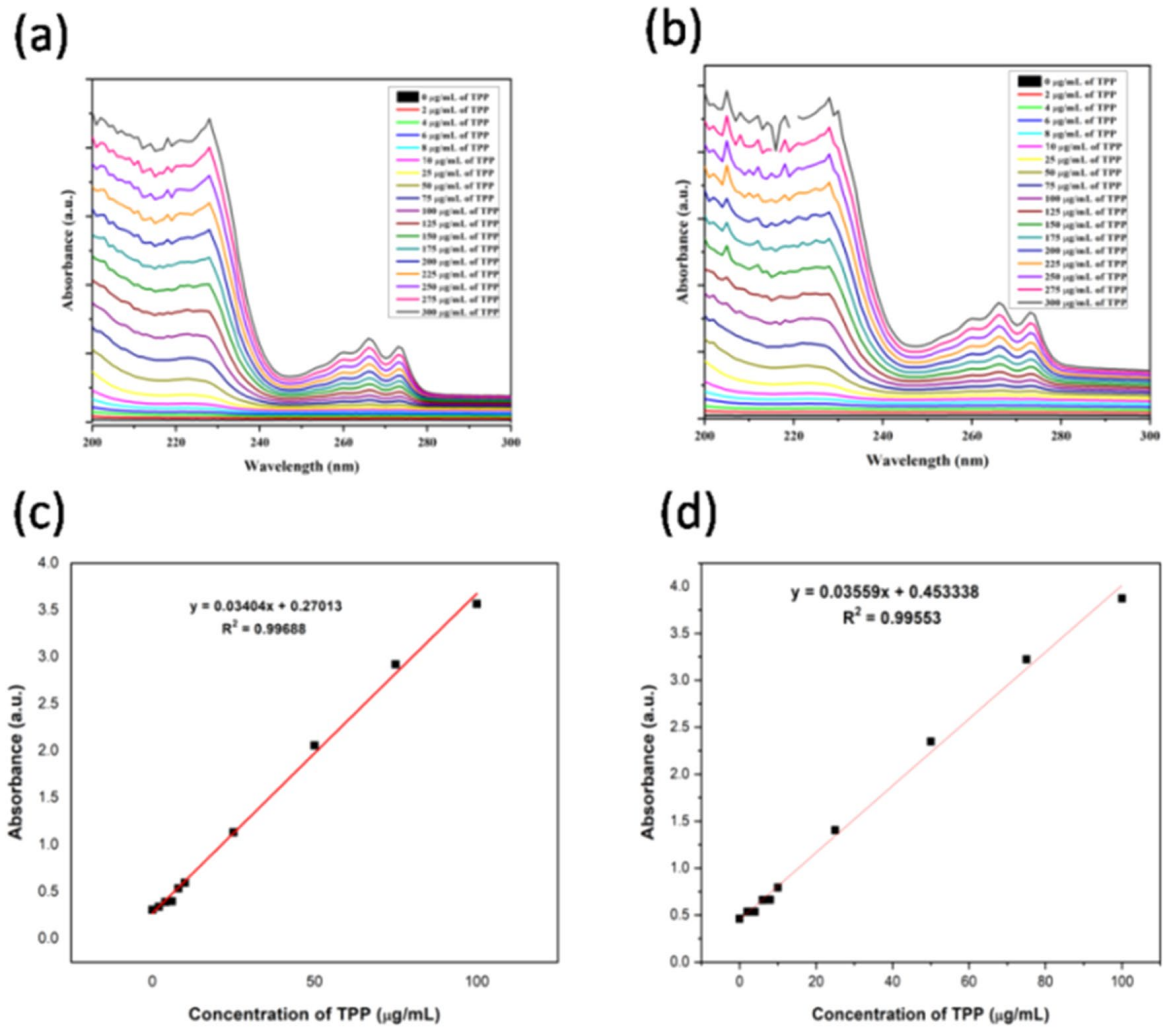
UV–*vis* absorption spectra of TPP conjugation on the surface of TPP-CTAC©AuNTs (**a**) and TPP-PSS@CTAC©AuNTs (**b**). A linear plot displaying absorbance of TPP measured at 229 nm upon conjugation with CTAC©AuNTs (**c**) and PSS@CTAC©AuNTs (**d**).

In this study, a zwitterion Ph_3_P^+^(CH_2_)_3_SH- (so-called, thiolated-TPP) was conjugated onto the surface of positive (CTAC©AuNTs) and negatively (PSS@CTAC©AuNTs) charged gold nanoparticles^43^. The conjugation of TPP onto the surface of CTAC©AuNTs and PSS@CTAC©AuNTs is monitored by UV–*vis* absorption spectroscopy (Fig. 3a,b). An apparent increase in peak upon increasing concentration of TPP (0–300 μg/mL) at 229 nm for both CTAC©AuNTs and PSS@CTAC©AuNTs infers successful conjugation^47,50^. Based on this, the loading efficacy (n = 3) of TPP on the surface of CTAC©AuNTs and PSS@CTAC©AuNTs was found to be 96.93% and 96.36% (Fig. 3c,d).

**Figure. 4:**
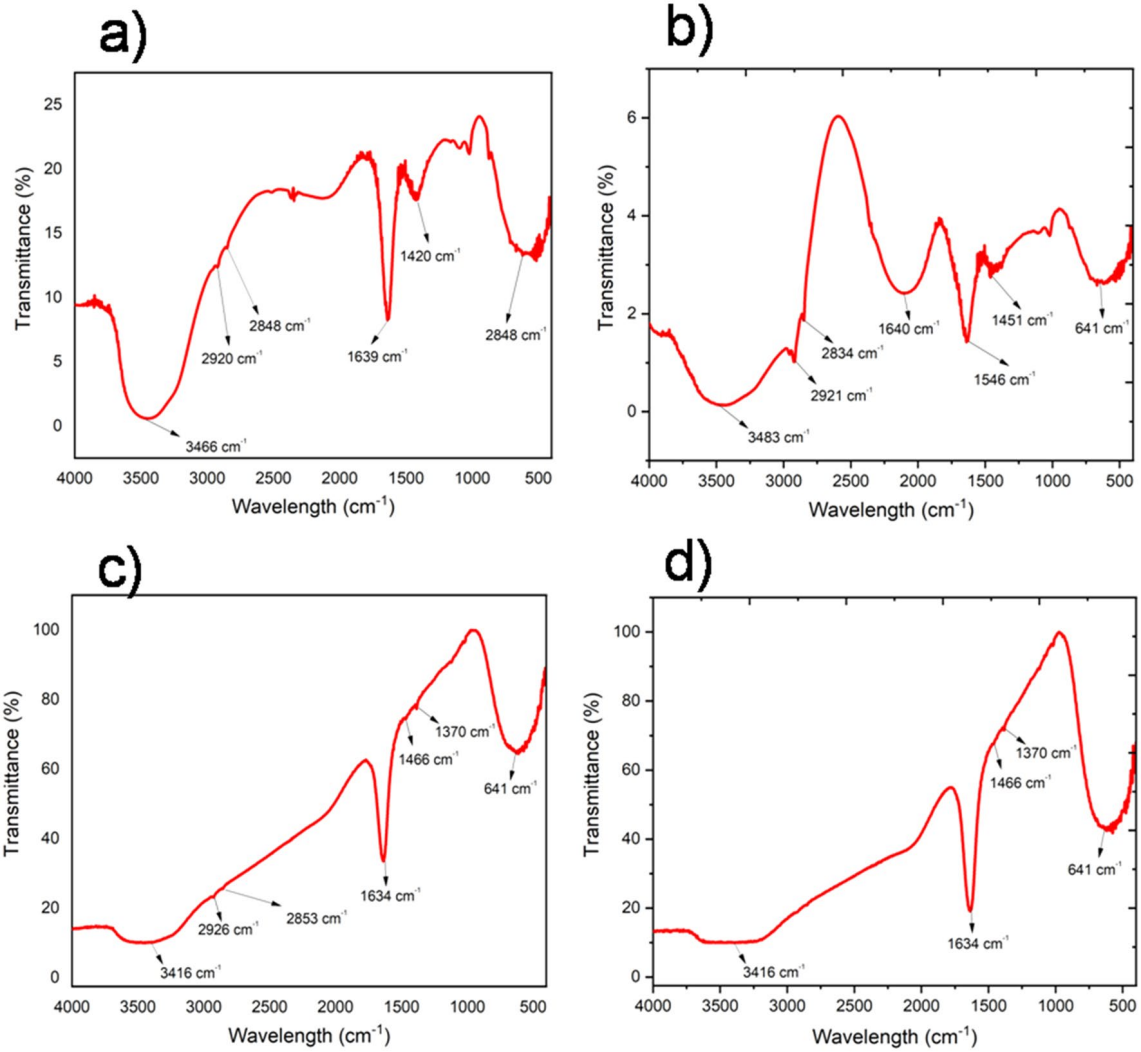
FTIR spectroscopic analysis. (**a**) CTAC©AuNTs. (**b**) PSS@CTAC©AuNTs. (**c**) TPP-CTAC©AuNTs. (**d**) TPP-PSS@CTAC©AuNTs.

Further, the conjugation of TPP onto the surface of CTAC©AuNTs and PSS@CTAC©AuNTs is ascertained by FTIR spectroscopy. For CTAC©AuNTs (Fig. 4a), the broadband at 3466 cm^−1^ can be assigned to –OH stretch in H-bonded water. A weak signals at 2920 and 2848 cm^−1^ an be ascribed to the –CH_2_ stretch of the CTAC chain^51^. The peaks observed between 1600 and 1400 cm^−1^ results from the deformation vibrations of –CH_3_ and CH_2_ groups. A strong peak at 720 cm^−1^ is designated to the existence of –CH_3_ and –CH_2_ groups^51^ on the surface of AuNTs. Upon conjugation with TPP (Fig. 4c), a reduction in the signature peaks for CTAC is noticed. In addition, authenticated peaks for TPP between 1600 and 1100 cm^−1^ corresponding to a carbonyl group (C=O) is observed, confirming the successful conjugation (Fig. 4c). SI, Fig. 1 shows the FTIR spectrum of TPP.

In the meantime, FTIR analysis claimed the existence of functional groups for PSS on the surface of CTAC©AuNTs (Fig. 4b). Based on the results, it is confirmed that the capping of CTAC and PSS on the surface of AuNTs provides precise interaction with TPP (Fig. 4d). Furthermore, a few characteristics peak unveiled by FTIR is in concise with earlier reports for the existence of PSS^52^ weak signals at 2921 and 2834 cm^−1^ designate the presence of CTAC with –CH_2_ stretch; peaks at 2106 and 1640 cm^−1^ infer the in-plane skeletal vibrations of benzene ring; the peaks at 1546 and 1451 cm^−1^ defines the symmetric and asymmetric vibrations of -SO_3_ group; the existence peaks at 669 cm^−1^ infers the existence of C-H aromatic stretching vibrations. However, the conjugation of TPP with PSS@CTAC©AuNTs is consistent with TPP-CTAC©AuNTs based on the peaks for the carbonyl group (C=O) between 1600 and 1100 cm^-1^.

**Figure. 5:**
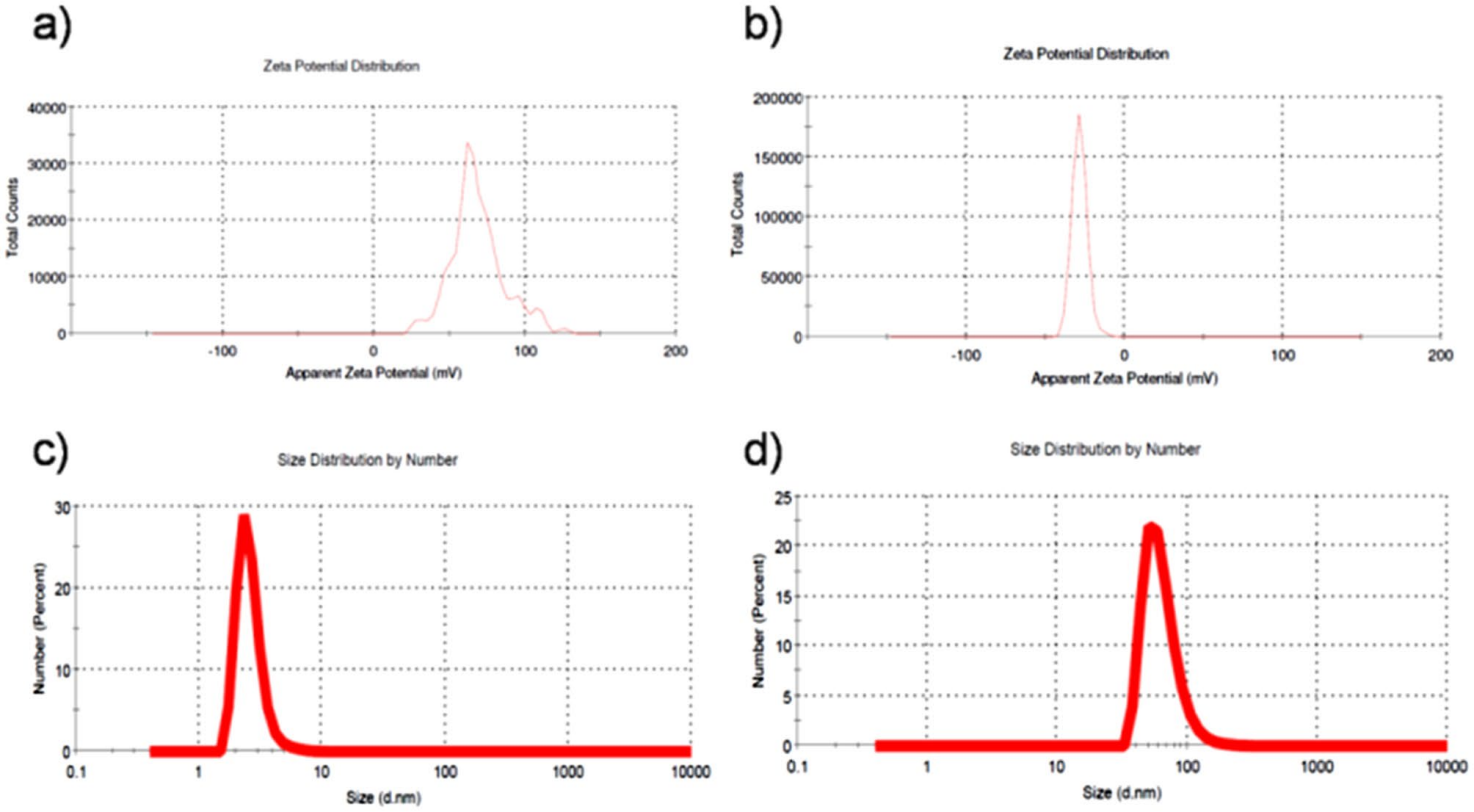
Dynamic light scattering measurements. Zeta potential for (**a**) TPP-CTAC©AuNTs (23.9 ± 3.93 mV and (**b**) TPP-PSS@CTAC©AuNTs (− 28.4 ± 4.93 mV). Particle size distribution analysis for (**c**) TPP-CTAC©AuNTs (48.50 ± 2.60 nm with PDI value of 0.324) and (**d**) TPP-PSS@CTAC©AuNTs (110.7 ± 2.41 nm with PDI value of 0.277).

We presume that, a non-covalent interaction (electrostatic and hydrogen bonding) between TPP and two different surface-charged AuNTs can exist^47,50^. Before conjugation, the surface charge of CTAC©AuNTs and PSS@CTAC©AuNTs was found to be + 33 ± 10.9 and − 42.9 ± 9.88 mV, respectively (Figs. 1h, 2h). However, a change in surface charge for both TPP-CTAC©AuNTs (23.9 ± 3.93 mV) and TPP-PSS@CTAC©AuNTs (− 28.4 ± 4.93 mV) (Fig. 5a,b) is noted inferring successful conjugation. Meanwhile, an increased hydrodynamic diameter assumes the contact of TPP with CTAC©AuNTs (48.50 ± 2.60 nm) and PSS@CTAC©AuNTs (110.7 ± 2.41 nm) (Fig. 5c,d). We also noticed that the TPP-CTAC©AuNTs and TPP-PSS@CTAC©AuNTs were stable in Milli-Q-water and PBS (SI, Table 2). Based on the above, it is clear that both TPP-CTAC©AuNTs and TPP-PSS@CTAC©AuNTs can be further taken for biomedical application.

**Figure 6:**
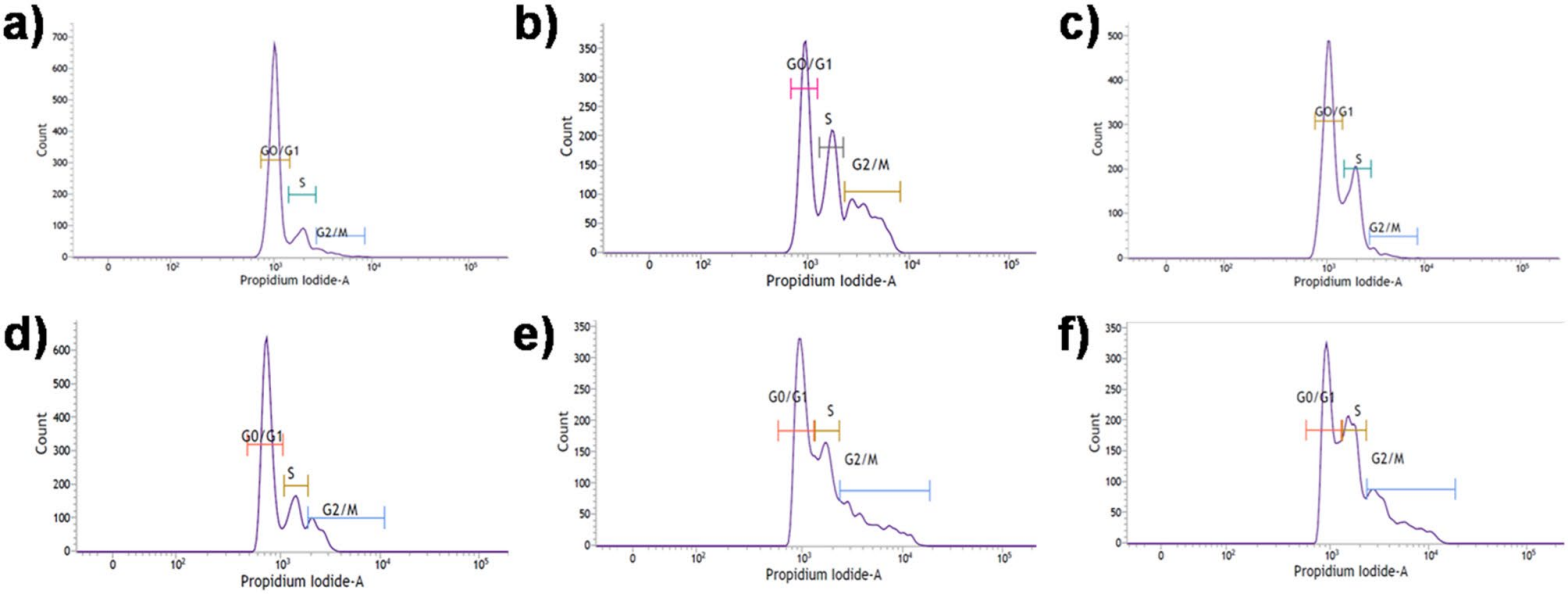
Cell cycle arrest induced by gold nanoconjugates in the presence of 5-ALA mediated PDT on breast cancer cells. MCF-7(a-c) and MDA-MB-231 (**d**–**f**) breast cancer cells. (**a**) Control (untreated cells). (b) IR-5-ALA-TPP-CTAC©AuNTs (IC_50_–0.71 ± 0.14* µg*/mL). (**c**) IR-5-ALA-TPP-PSS@CTAC©AuNTs (IC_50_-0.67 ± 0.89 µg/mL). (**d**) Control (untreated cells). (**e**) IR-5-ALA-TPP-CTAC©AuNTs (IC_50_-0.78 ± 0.55 µg/mL). (**f**) IR-5-ALA-TPP-PSS@CTAC©AuNTs (IC_50_- 0.58 ± 0.23* µg*/mL).

### Cytotoxicity assay

To assess the cell viability of CTAC©AuNTs, PSS@CTAC©AuNTs, TPP, TPP-CTAC©AuNTs, and TPP-PSS@CTAC©AuNTs were subjected to MTT (3-[4,5-dimethylthiazol-2-yl]-2,5 diphenyl tetrazolium bromide) assay to measure the total mitochondrial activity, which is directly proportional to the number of viable cells^47,50^. In our study, all the compounds caused dose-dependent cell death in MCF-7 and MDA-MB-231 breast cancer cell lines (SI, Table 3). TPP-CTAC©AuNTs had a higher cytotoxicity than TPP-PSS@CTAC©AuNTs > CTAC©AuNTs > PSS@CTAC©AuNTs > TPP. Furthermore, the cytotoxicity of the above compounds against normal human embryonic cells (HEK-293) is lower than that of cancer cells, as shown in SI, Table 3. It is also noted that nanomaterial's surface charge might play a pivotal role in killing breast cancer cells^53,54^. Based on the results, positively charged materials were more cytotoxic than negatively charged components^43^.

### Cellular PDT measurement

The PDT effect of TPP-CTAC©AuNTs and TPP-PSS@CTAC©AuNTs against normal (HEK-293) and cancer cells of the breast (MCF-7 and MDA-MB-231) was measured by incubating the cells in media containing 0.5 mM 5-ALA solution^43^. Later, the media containing 0.5 mM 5-ALA was thoroughly removed, followed by the treatment with CTAC©AuNTs, PSS@CTAC©AuNTs, TPP, TPP-CTAC©AuNTs, and TPP-PSS@CTAC©AuNTs. As a prerequisite for PDT therapy, the cells were immediately exposed to a halogen light for 1 min before being incubated in a CO_2_ incubator for evaluating cytotoxicity by MTT assay after 24 h. Further, the MTT assay confirmed that the irradiating cells with TPP-CTAC©AuNTs and TPP-PSS@CTAC©AuNTs exposed to 5-ALA (0.5 mM) showed increased cytotoxicity compared to non-radiated cells (SI, Table 4). In addition, the effect of IR-TPP-CTAC©AuNTs and IR-TPP-PSS@CTAC©AuNTs against normal cells (HEK-293) was found to be minimal. The study also noted that IR-TPP-PSS@CTAC©AuNTs have a substantially better killing effect than IR-TPP-CTAC©AuNTs. Our findings are consistent with previous reports on surface charge-based PDT measurement^32,43^. On the contrary, the exact killing mechanism of IR-TPP-CTAC©AuNTs and IR-TPP-PSS@CTAC©AuNTs requires further investigation.

### Morphological analysis utilizing fluorescent probes

**Figure 7:**
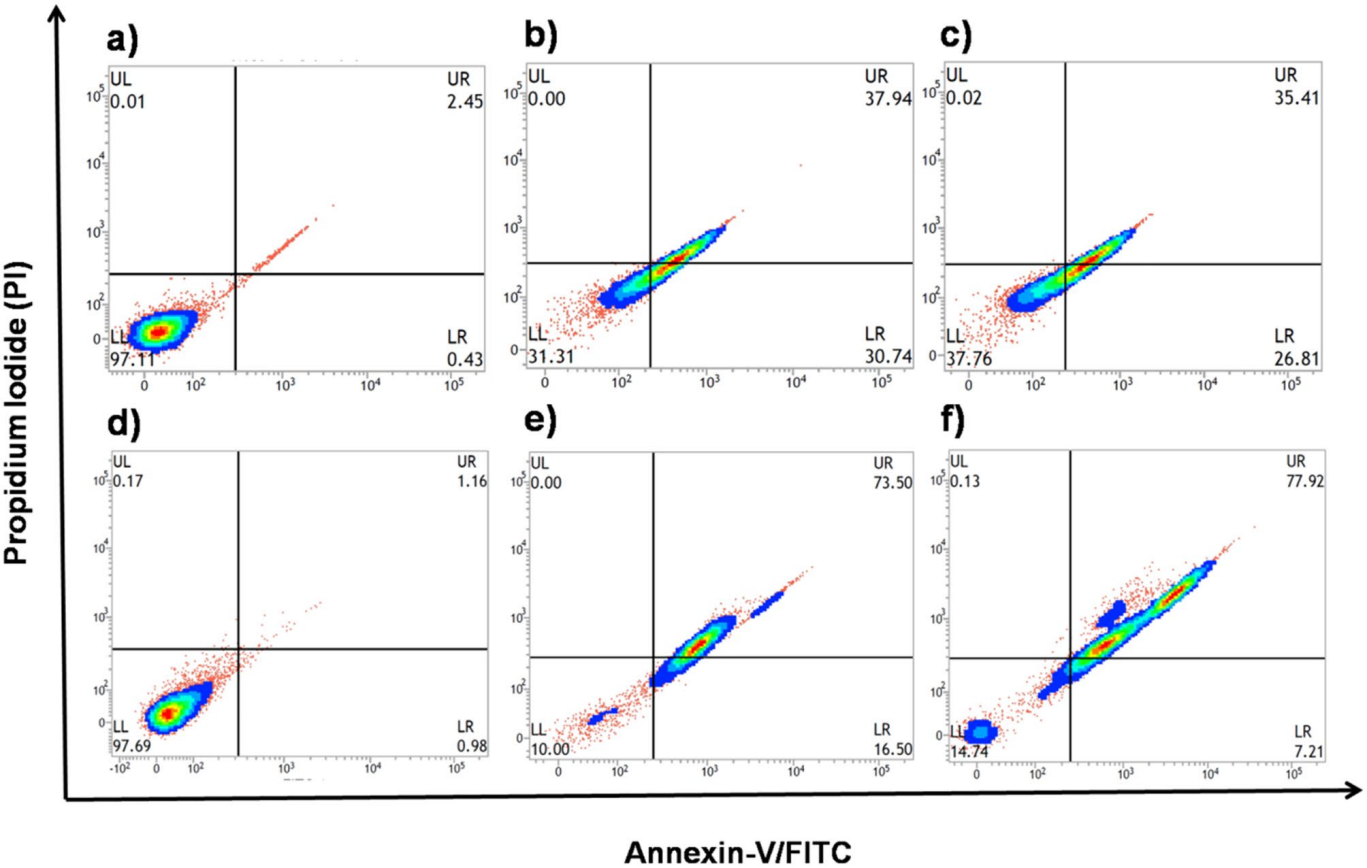
Effect of gold nanoconjugates in the presence of 5-ALA mediated PDT targeting MCF-7 (a-c) and MDA-MB-231 (**d**–**f**) breast cancer cells. (**a**) Control (untreated cells). (**b**) IR-5-ALA-TPP-CTAC©AuNTs (IC_50_–0.71 ± 0.14 µg/mL). (**c**) IR-5-ALA-TPP-PSS@CTAC©AuNTs (IC_50_-0.67 ± 0.89 µg/mL). (**d**) Control (untreated cells). (**e**) IR-5-ALA-TPP-CTAC©AuNTs (IC_50_-0.78 ± 0.55 µg/mL). (**f**) IR-5-ALA-TPP-PSS@CTAC©AuNTs (IC_50_-0.58 ± 0.23 µg/mL).

**Figure 8:**
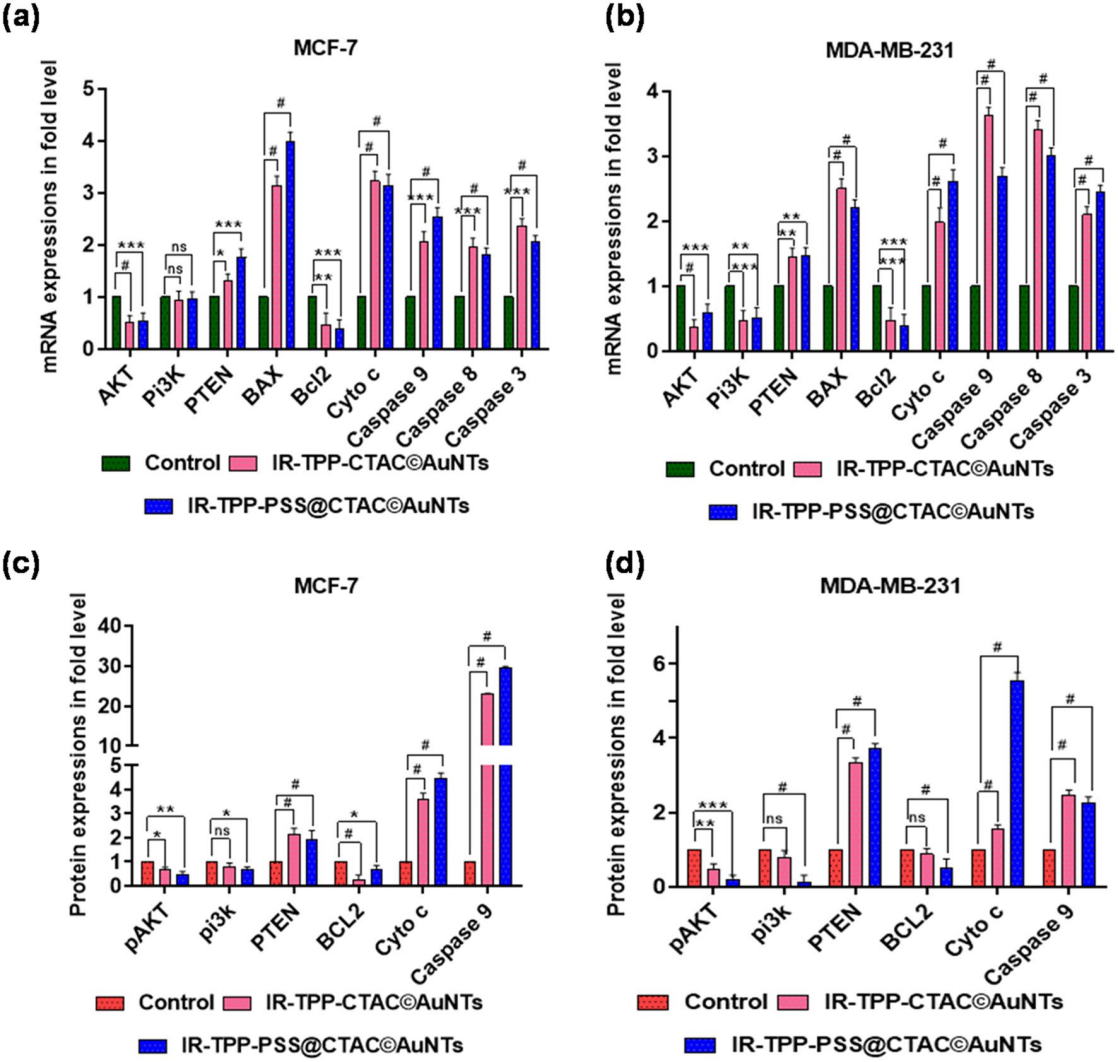
Representative graphs of semi-quantitative RT-PCR (**a**, **b**) and western blot analysis (**c**, **d**) showing the effects of gold nanoconjugates in the presence of 5-ALA mediated PDT on breast cancer cell in fold changes. All quantified values are represented as mean ± SD (n = 3). Statistical significance was performed by one-way ANOVA followed by Dunnett's multiple test. Values are statistically significant at ^*#*^*p* < 0.0001, ^***^p < 0.001, ^**^p < 0.01, ^*^p < *0.05, and ns.*

To investigate the effect of IR-TPP-CTAC©AuNTs and IR-TPP-PSS@CTAC©AuNTs, we performed various staining methods to study the basic morphological changes of apoptosis, nuclear fragmentation, generation of ROS, and mitochondrial membrane permeation in breast cancer cells (MCF-7 and MDA-MB-231) (SI, Figs. 2–6). We found no substantial morphological changes in control (untreated) cells employed in our investigation. However, the induction of apoptosis is revealed by live/dead cells with green and red fluorescence (SI, Fig. 2). On the other hand, investigations on nuclear fragmentation exhibit blue fluorescence, implying chromatin condensation with irreversible pyknotic nuclei (SI, Fig. 3, 4). ROS measurements support the reduction in green fluorescence owing to an increased oxidative stress environment^50,47^ (SI, Fig. 5). Mitochondrial membrane potential study implies decay in green fluorescence that hampers electron transport and oxidative phosphorylation^55^ (SI, Fig. 6). Based on the findings, we detected all conceivable apoptotic events, such as fragmented nuclei, increased ROS production, and mitochondrial membrane potential, after treating two breast cancer cells (MCF-7 and MDA-MB-231) with IR-TPP-CTAC©AuNTs and IR-TPP-PSS@CTAC©AuNTs in the presence of 5-ALA mediated PDT. Experiments with CTAC©AuNTs, PSS@CTAC©AuNTs, TPP-CTAC©AuNTs, and TPP-PSS@CTAC©AuNTs show similar results with a half maximum inhibitory concentration (IC_50_) (SI, Figs. 7–11). We presume that irradiation by PDT in the presence of 5-ALA improves the targeting of breast cancer cells by inducing apoptosis. Moreover, the morphological examination should be correlated well with gene and protein expression studies.

### PpIX measurements

The present study utilizes a photo-sensitizer, (5-ALA) that induces PDT in breast cancer cells upon irradiation with IR-TPP-CTAC©AuNTs and IR-TPP-PSS@CTAC©AuNTs as shown in SI, Fig. 12. As a result, cells produce more singlet oxygen than those treated cells without 5-ALA. It has been reported that, the increased singlet oxygen production is related to the conversion of 5-ALA into protoporphyrin IX (PpIX), that occurs majorly in mitochondria^43^. It is also reported that, the generation of singlet oxygen will directly influence cell death by two indicators, viz. endogenous (mediated by loss of mitochondrial membrane potential) and exogenous (mediated by endoplasmic reticulum) mechanism^56^. Considering the fact, the mitochondrial targeting agent (TPP) accumulates well within mitochondria leading to enhanced membrane permeability^57^.

### Co-localization experiments

By utilising spectral deconvolution, the co-localization of molecules within intracellular compartments can be successfully explored. In light of this, the co-localization of TPP in MCF-7 and MDA-MB-231 breast cancer cells was investigated using Raman spectroscopy, as depicted in SI, Fig. 13. Upon exposure of cells with TPP, a band in the region at 900–1200 cm^−1^ is attributed to Ph-P vibrations^43^. Similarly, breast cancer cells treated to TPP-CTAC©AuNTs and TPP-PSS@CTAC©AuNTs exhibited identical signature peaks, indicating the co-localization of nanoprobes. It is also noticed that the signals from TPP-CTAC©AuNTs and TPP-PSS@CTAC©AuNTs were weak, suggesting that TPP and AuNTs were conjugated. The study's findings are consistent with previous reports on the co-localization of nanomaterials using Raman spectroscopy^58,59^.

### Cell cycle

Flow cytometric analysis of MCF-7 and MDA-MB-231 breast cancer cell cycle pattern upon treatment with 5-ALA-based PDT in the presence of IR-TPP-CTAC©AuNTs and IR-TPP-PSS@CTAC©AuNTs AuNTs is shown in Fig. 6. MCF-7 cells treated with IR-TPP-CTAC©AuNTs and IR-TPP-PSS@CTAC©AuNTs exhibited a G0/G1 cell cycle arrest of 64.95 and 79.26%, respectively, compared to untreated cells^60^. Meanwhile, IR-TPP-CTAC-AuNTs and IR-TPP-PSS@CTAC-AuNTs showed similar G0/G1 phase cycle arrest outcomes in MDA-MB-231 breast cancer cells, with 44.88 and 40.13%, respectively. The study found that 5-ALA-based PDT treatment inhibited G0/G1 phase in breast cancer cells by accumulating cell populations after 24 h in the presence of IR-TPP-CTAC©AuNTs and IR-TPP-PSS@CTAC©AuNTs (SI, Fig. 14).

### Annexin V-FITC/Propidium Iodide

In the present study, flow cytometric analysis was used to validate the progression of cell death following 5-ALA-based PDT^61^. Annexin-V-FITC has been demonstrated to detect phosphatidyl-serine (PS) exposed on the cell surface during apoptosis, which is an early sign. After 24 h of exposure, IR-TPP-CTAC©AuNTs and IR-TPP-PSS@CTAC©AuNTs (at IC_50_ values) caused apoptosis in breast cancer cells, which is consistent with prior studies on morphological characteristics in breast cancer cells. As depicted in Fig. 7, the progression of apoptosis was evident in the dot plots. According to flow cytometric data, PDT with 5-ALA induce apoptosis in approximately 60, and 90 percent of MCF-7 and MDA-MB-231 breast cancer cells^62^ (SI, Fig. 15).

### Molecular mechanism

In recent years, drug delivery systems have been developed to target specific organs or tissues with the desired therapeutic concentration^63,64^. However, targeting cancer with TPP-functionalized gold nanoprobes is limited (TPP-CTAC©AuNTs). Taking this into consideration, we engineered TPP functionalized gold nanoparticles with two different surface charges (IR-TPP-CTAC©AuNTs and IR-TPP-PSS@CTAC©AuNTs) and evaluated its PDT efficacy in the presence of 5-ALA in breast cancer cells (MCF-7 and MDA-MB-231) via the inhibition of Pi3k/Akt signaling pathway^65,66^.

### Activation of PTEN in Pi3K/Akt signaling

The Pi3K/AKT/mTOR pathway regulates an extensive array of biological functions, including cell growth, proliferation, metabolism, and angiogenesis^67^. However, Phosphatase and tensin (PTEN) protein homolog activation can negatively regulate the Pi3K-AKT pathway^68^. As a result of PTEN activation, the downstream signaling events are hindered in their ability to protect tumor development. Thus, the Pi3K/AKT pathway may be an ideal target for cancer therapeutic intervention^69^. In view of this, we examined the gene and protein expression of Pi3K, AKT, and PTEN in IR-TPP-CTAC©AuNTs and IR-TPP-PSS@CTAC©AuNTs treated breast cancer cells (MCF-7 and MDA-MB-231) (Fig. 8). The gene and protein expression pattern of Pi3K, AKT and PTEN is shown in SI, Fig. 16, 17.

In two breast cancer (MCF-7 and MDA-MB-231) cells treated with IR-TPP-CTAC©AuNTs and IR-TPP-PSS@CTAC©AuNTs, the Pi3K/AKT gene expression was significantly reduced (Fig. 8)^70^. Compared to untreated cells, the tumor suppressor gene PTEN was up-regulated considerably, thereby regulating cell proliferation. A western blot study in breast cancer cell lines revealed that the Pi3K/AKT pathway was down-regulated (Fig. 8). In contrast, PTEN expression was up-regulated, associated with the deregulation of growth and extracellular signals (Fig. 8)^71^. Hence, it is clear from the study that 5-ALA-based PDT in the presence of IR-TPP-CTAC©AuNTs and IR-TPP-PSS@CTAC©AuNTs significantly down-regulate Pi3K/AKT pathway leading to the apoptotic events^72^.

### Targeting mitochondrial membrane permeability

The permeability of the mitochondrial membrane during apoptosis is controlled by the Bcl-2 family of proteins, which may be anti-apoptotic or pro-apoptotic^73^. For example, Bax (pro-apoptotic) protein is primarily found in the cytoplasm, while Bcl-2 (anti-apoptotic) is located in the nucleus and mitochondria. However, during the progression of apoptosis, Bax gets triggered and travels mitochondria by hampering the expression of Bcl-2^74^. As a result, cytochrome c is released, leading to a decrease in mitochondrial membrane potential^75^. Thus, the expression pattern of anti-apoptotic (Bcl-2) and pro-apoptotic (Bax & cytochrome c) needs to be addressed upon treatment with IR-TPP-CTAC©AuNTs and IR-TPP-PSS@CTAC©AuNTs (Fig. 8).

In our study, the gene and protein expression patterns of Bcl-2 were considerably down-regulated in both cancer cells after treatment with IR-TPP-CTAC©AuNTs and IR-TPP-PSS@CTAC©AuNTs^76^ In addition, the gene expression level of Bax was elevated, confirming the mitochondrial membrane potential has decreased. Meanwhile, a high Bax/Bcl-2 ratio has been hypothesized to trigger the breakdown of the mitochondrial membrane potential, releasing cytochrome c and inducing cell death^77^. Our findings also demonstrate that reduced Bcl-2 protein expression and increased Bax protein expression results in cytochrome c activation. The activation of cytochrome c from mitochondria is a crucial beginning step in the process of apoptosis^78^. Figure 8 depicts the elevation of cytochrome c gene and protein expression levels in breast cancer cells treated with IR-TPP-CTAC©AuNTs and IR-TPP-PSS@CTAC©AuNTs (SI, Fig. 16, 17).

### Role of caspase in apoptosis induction

During apoptosis, mitochondrial membranes become permeable, allowing cytochrome c to enter the cytoplasm and activate caspases through oligomerization of the adaptor molecule apoptosis-protease activating factor 1 (Apaf-1) called apoptosomes^79^. Each apoptosome recruits seven dimers of caspase-9, favouring the activation of caspase-3, leading to intrinsic apoptosis^80^. Meanwhile, the extrinsic pathway of apoptosis triggers the death-inducing signaling complex (DISC) by activating a plethora of signaling events leading to the activation of caspase-8^81^. As a result, active caspase-9 gets activated, mobilzing downstream caspases (Caspase-3) and thereby initiating apoptosis^82^.

In our study, treatment with IR-TPP-CTAC©AuNTs and IR-TPP-PSS@CTAC©AuNTs increases mRNA expression patterns for caspase-9, caspase-8, and caspase-3 (Fig. 8). This helps us understand the activation of both intrinsic and extrinsic cell death signals in breast cancer cells (MCF-7 and MDA-MB-231)^83–85^. In addition, the protein expression pattern cleaved caspase-9 was significantly over-expressed and coincides with the induction of apoptosis, as revealed by western blot analysis upon treatment with IR-TPP-CTAC©AuNTs and IR-TPP-PSS@CTAC©AuNTs treated cells following 5-ALA-based PDT than control cells (SI, Fig. 16, 17).

On the whole, 5-ALA-based PDT activates the tumor suppressor gene (PTEN) in IR-TPP-CTAC©AuNTs IR-TPP-PSS@CTAC©AuNTs treated cells, inhibiting Pi3K/AKT signaling. In addition, it is also noticed from the study that 5-ALA-based PDT in combination with IR-TPP-CTAC©AuNTs and IR-TPP-PSS@CTAC©AuNTs may lead to the activation of mitochondrial-mediated apoptosis by the down-regulation of anti-apoptotic and upregulation of pro-apoptotic (BAX, cytochrome-c, caspase-8, 9 and 3) entities.

## Conclusion

We have developed surface charge optimized nanoconjugates system that impairs cell survival Pi3K/AKT signaling. The nanoconjugates system comprise of anionic and cationic AuNTs conjugated with TPP containing S–H group. The surface charge optimized AuNTs conjugated with TPP offers cytotoxicity upon 5-ALA based PDT treatment. As a result, induction of apoptosis occurs via DNA damage; generate ROS species and deregulate mitochondria. The combined effect of 5-ALA and PDT with gold nanoconjugates persuades apoptosis in breast cancer cells. We believe that, the proposed gold nanoconjugates system is a promising anti-cancer agent that targets mitochondria leading to cellular apoptosis in breast cancer.

## Supplementary Information

Supplementary Information.

## Data Availability

The datasets used and/or analyzed during the current study are available from the corresponding author on reasonable request.
