# Supplementary material for: Triphenylphosphonium conjugated gold nanotriangles impact Pi3K/AKT pathway in breast cancer cells: a photodynamic therapy approach
Source: Sci Rep. 2023 Feb 8;13:2230. doi: 10.1038/s41598-023-28678-x (PMC9908940; doi:10.1038/s41598-023-28678-x)

**SUPPORTING INFORMATION**

**Triphenylphosphonium conjugated gold nanotriangles impact Pi3K/AKT pathway in breast cancer cells: a photodynamic therapy approach**

Nadar Manimaran Vinita^1^, Umapathy Devan^2^, Sabapathi Durgadevi^1^, Selvaraj Anitha^1^, Dhamodharan Prabhu^3^, Sundarraj Rajamanikandan^3,9^, Muthusamy Govarthanan^4,5^, Ananthanarayanan Yuvaraj^6^, Muniyandi Biruntha^1^, Arockiam Antony Joseph Velanganni^2^, Jeyaraman Jeyakanthan^7^, Pitchan Arul Prakash^8^, Mohamed Sultan Mohamed Jaabir^8^, Ponnuchamy Kumar^1,*^

^1^Food Chemistry and Molecular Cancer Biology Lab, Department of Animal Health and Management, Alagappa University, Karaikudi-630 003, Tamil Nadu, India

^2^Molecular Oncology Laboratory, Department of Biochemistry, Bharathidasan University, Tiruchirappalli – 620 024, Tamil Nadu, India

^3^Research and Development Wing, Sree Balaji Medical College and Hospital (SBMCH), Bharath Institute of Higher Education and Research (BIHER), Chrompet, Chennai – 600 044, Tamil Nadu, India

^4^Department of Environmental Engineering, Kyungpook National University, Deagu, 41566, Republic of Korea.

^5^Department of Biomaterials, Saveetha Dental College and Hospital, Saveetha Institute of Medical and Technical Sciences, Chennai – 600077, Tamil Nadu, India

^6^Department of Zoology, Periyar University, Salem - 636 011, Tamil Nadu, India

^7^Department of Bioinformatics, Alagappa University, Karaikudi – 630 003, Tamil Nadu, India

^8^PG and Research Department of Biotechnology and Microbiology, The National College, Tiruchirappalli – 620 001, Tamil Nadu, India.

^9^Department of Biochemistry, Centre for Drug Design, Karpagam Academy of Higher Education, Coimbatore – 641 021, Tamil Nadu, India.

***Corresponding author**

Ponnuchamy Kumar, Food Chemistry and Molecular Cancer Biology Lab, Department of Animal Health and Management, Alagappa University, Karaikudi-630 003, Tamil Nadu, India. Email: kumarp@alagappauniversity.ac.in

**SI, Table 1.**  Primers used in the Study

| **Gene** | **Primers used** |
| --- | --- |
| AKT | F - 5’- CAGTGGACCACCTTCGTTGA - 3’  R - 5'- ACAGAGTCGGCCACTGATTG - 3' |
| PI3K | F - 5’- GGAAGCCCTCCAGAAAGGTC - 3’  R - 5’- GCACTCGGAAGTTGAATGGC - 3’ |
| PTEN | F - 5'- TCCCAGACATGACAGCCATC - 3'  R - 5’- TGTCTTTCAGCACAACTTACTACA - 3’ |
| BAX | F - 5’- AGCAAACTGGTGCTCAAGGC - 3’  R - 5’- CAGGGACATCAGTCGCTTCAG - 3’ |
| Bcl-2 | F - 5’- F-CTTTGAGTTCGGTGGGGTCA - 3’  R - 5’- GGGCCGTACAGTTCCACAAA - 3’ |
| Cytochrome c  (Cyto-c) | F - 5’- ACAAAGGCATCATCTGGGGAG - 3’  R - 5’- AGGCAGTGGCCAATTATTACTC - 3’ |
| Caspase-9 | F - 5'- TGAGACCCTGGACGACATCT - 3'  R - 5’- TCCCTTTCACCGAAACAGCA - 3’ |
| Caspase-8 | F - 5'- GCGGAGGGTCGATCATCTAT - 3'  R - 5'- TCCTTCTCCCAGGATGACCC - 3' |
| Caspase-3 | F - 5’- GTGCTATTGTGAGGCGGTTG - 3’  R - 5’- TCCAGAGTCCATTGATTCGCTT - 3’ |
| β-actin | F – 5’ – TGGAACGGTGAAGGTGACAG - 3’  R – 5’ – AACAACGCATCTCATATTTGGAA - 3’ |

**SI, Table 2. Comparision of Zeta Potential value of nanoprobes in milliQwater and PBS**

| **S.No** | **Nanoprobes** | **In MilliQ water** | **In PBS*** |
| --- | --- | --- | --- |
| 1 | CTAC AuNTs | + 33 ± 2.9 mV | 32.1 ± 2.38 mV |
| 2 | PSS@CTAC©AuNTs | - 42.9 ± 9.88 mV | - 46.6 ± 4.14 mV |
| 3 | TPP-CTAC AuNTs | 23.9 ± 3.93 mV | 22.5 ± 2.32 mV |
| 4 | TPP-PSS@CTAC©AuNTs | - 28.4 ± 4.93 mV | - 31.2 ± 3.84 mV |
| *Note the Zeta value of PBS is - 5.88 ± 1.36 mV | | | |

**SI, Table 3. MTT assay**

| **S. No** | **Cell lines** | **IC_50_ value (*µ*g/mL)** | | | |
| --- | --- | --- | --- | --- | --- |
|  |  | **CTAC©AuNTs** | **PSS@CTAC©AuNTs** | **TPP-CTAC©AuNTs** | **TPP-PSS@CTAC©AuNTs** |
| 1 | MCF-7 | 1.70 ± 0.23 | 1.99 ± 0.30 | 1.31 ± 0.11 | 1.29 ± 0.11 |
| 2 | MDA-MB-231 | 2.47 ± 0.39 | 2.24 ± 0.35 | 2.19 ± 0.34 | 1.74 ± 0.24 |
| 3 | HEK-293 | 12.65 ± 0.64 | 13.64 ± 0.65 | 11.05 ± 0.54 | 10.36 ± 0.74 |

**SI, Table 4. MTT assay**

| **S. No** | **Cell lines** | **IC_50_ value (*µ*g/mL)** | | | |
| --- | --- | --- | --- | --- | --- |
|  |  | **Non-irradiated** | | **Irradiated (IR)** | |
|  |  | **5-ALA-TPP-CTAC©AuNTs** | **5-ALA-TPP-PSS@CTAC©AuNTs** | **5-ALA-TPP-CTAC©AuNTs** | **5-ALA-TPP-PSS@CTAC©AuNTs** |
| 1 | MCF-7 | 1.09 ± 0.04 | 1.14 ± 0.57 | 0.71 ±-0.14 | 0.67 ± 0.89 |
| 2 | MDA-MB-231 | 1.38 ±0.14 | 1.52 ± 0.18 | 0.78 ± 0.55 | 0.58 ± 0.23 |
| 3 | HEK-293 | 8.64 ± 0.47 | 8.25 ± 0.45 | 6.456 ± 0.78 | 7.62 ± 0.38 |

**
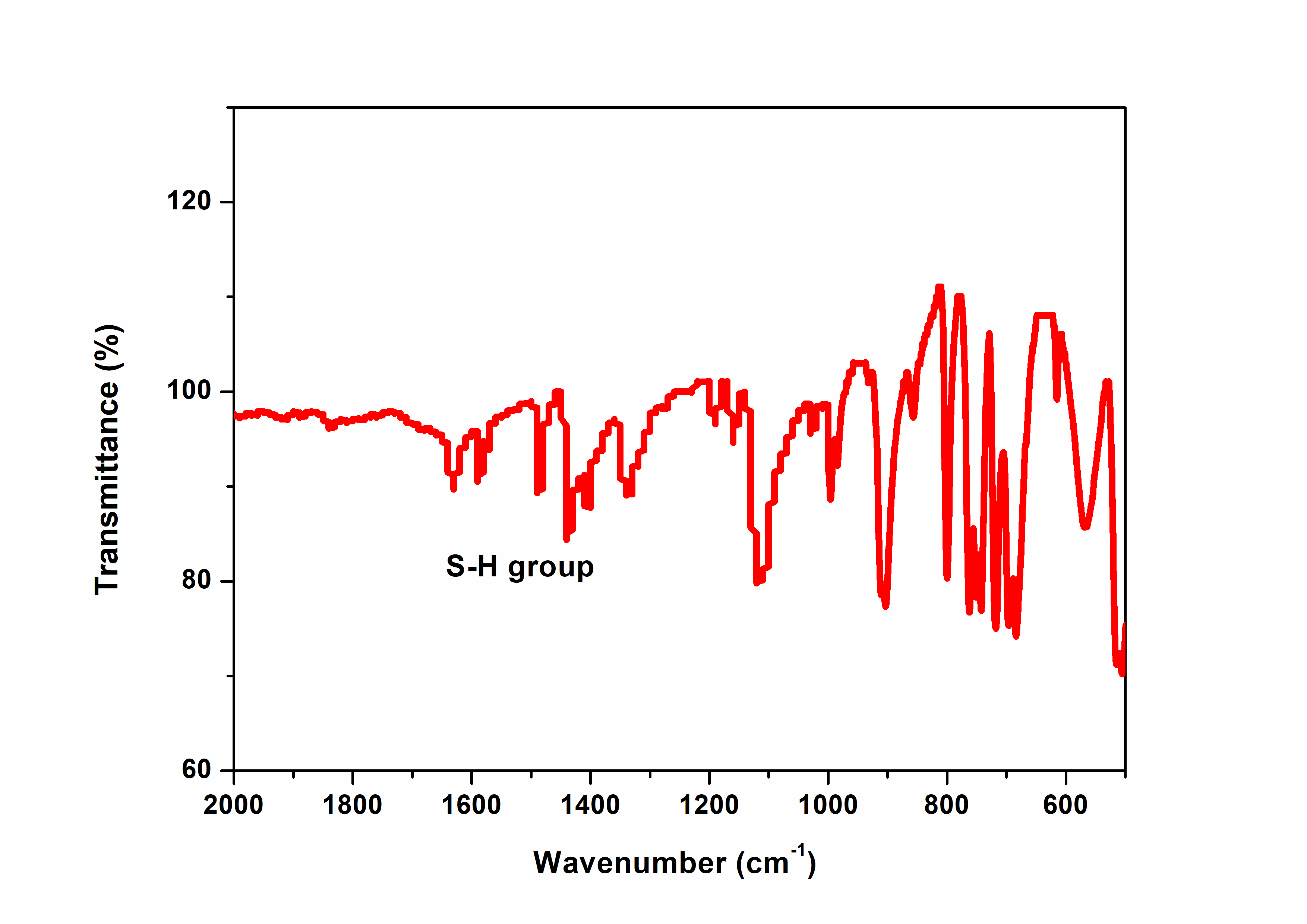
**

**SI, Figure 1.** FTIR analysis of TPP with thiol (S-H) group

**
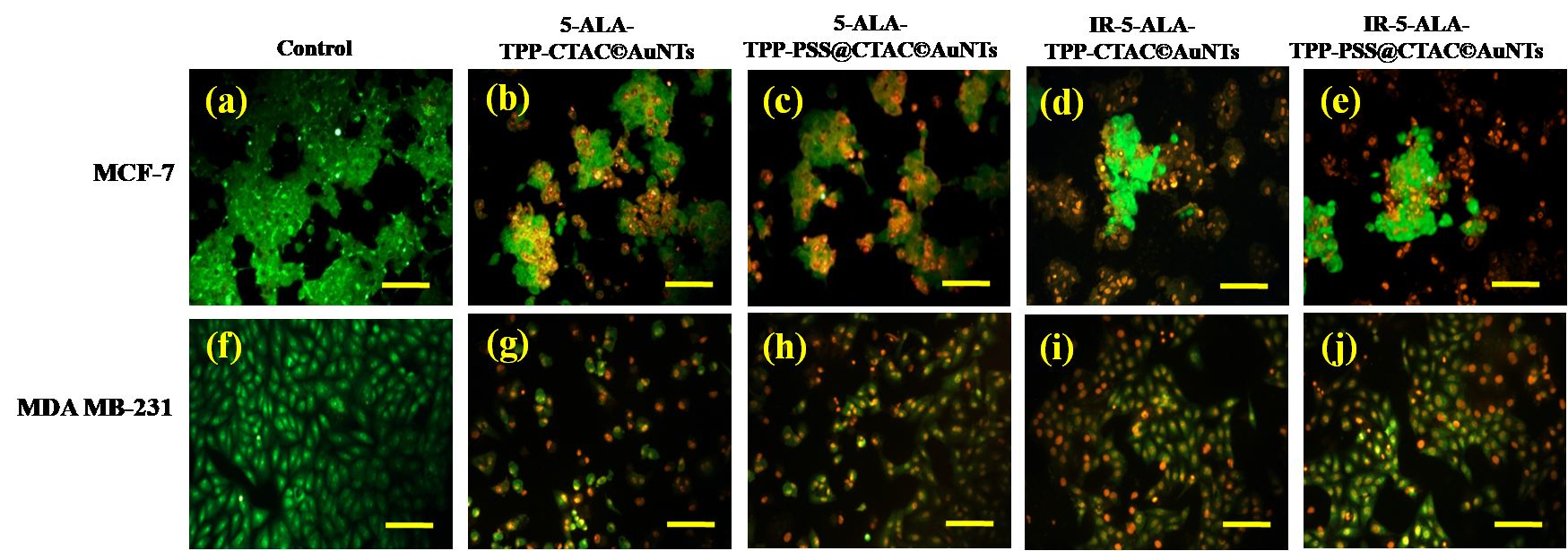
**

**SI, Figure 2.** Dual staining (AO/EtBr) on breast cancer cells (MCF-7 and MDA-MB-231) after treatment with gold nanoconjugates with and without irradiation in the presence of 5-ALA. (a,f) control (untreated cells). (b,g) 5-ALA-TPP-CTAC©AuNTs. (c,h) 5-ALA-TPP-PSS@CTAC©AuNTs. (d,i) IR-5-ALA-TPP-CTAC©AuNTs. (e,j) IR-5-ALA-TPP-PSS@CTAC©AuNTs. AO stains both live and dead cells in green, and EtBr stains DNA that undergo apoptosis. IR refers to irradiation. Scale bar – 125 µm/ 20× magnification).

**
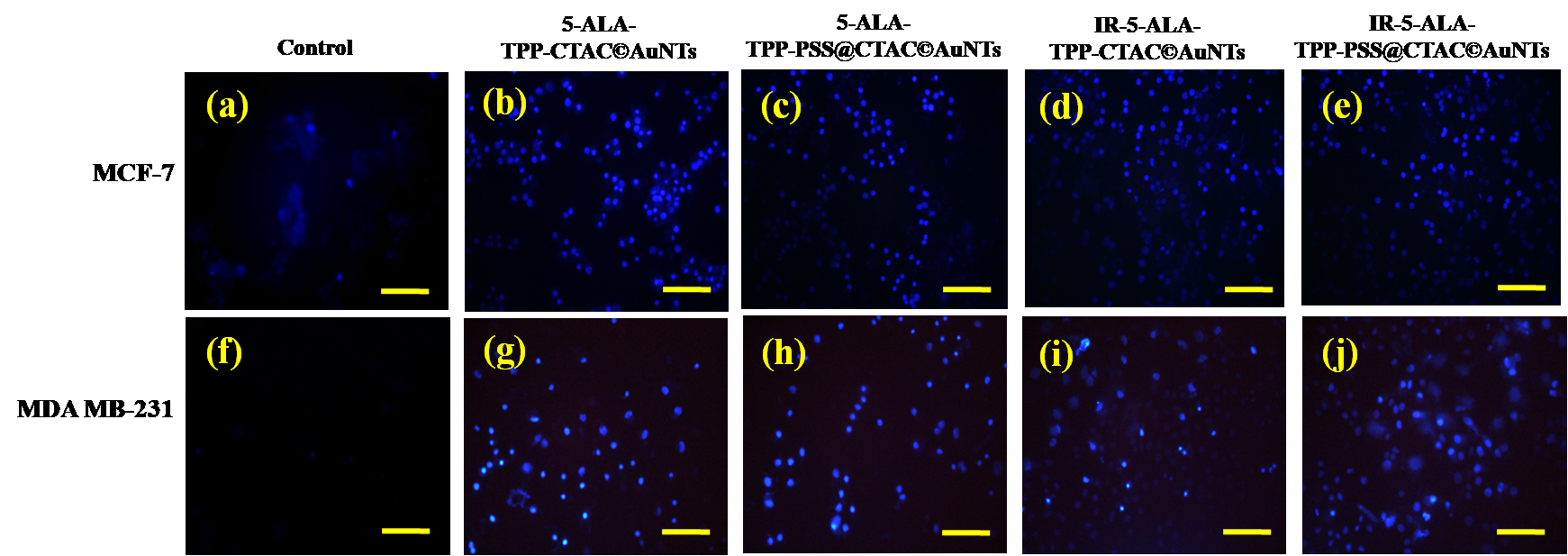
**

**SI, Figure 3.** Nuclear staining (Hoechst 33344) on breast cancer cells (MCF-7 and MDA-MB-231) after treatment with gold nanoconjugates with and without irradiation in the presence of 5-ALA. (a,f) control (untreated cells). (b,g) 5-ALA-TPP-CTAC©AuNTs. (c,h) 5-ALA-TPP-PSS@CTAC©AuNTs. (d,i) IR-5-ALA-TPP-CTAC©AuNTs. (e,j) IR-5-ALA-TPP-PSS@CTAC©AuNTs. Hoechst 33344 stains AT-rich regions of DNA whose cell membrane is damaged. In control, cells show no blue emission, inferring no cell death. IR refers to irradiation. Scale bar – 125 µm/ 20× magnification).


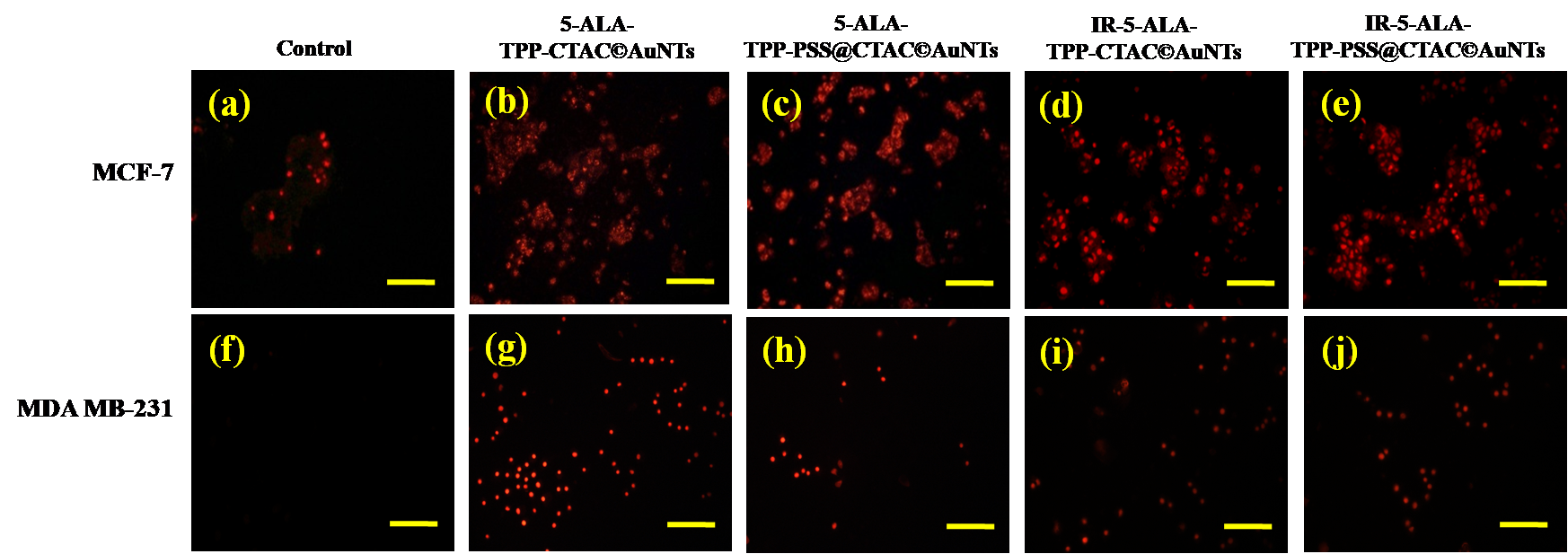


**SI, Figure 4.** DNA staining (PI) on breast cancer cells (MCF-7 and MDA-MB-231) after treatment with gold nanoconjugates with and without irradiation in the presence of 5-ALA. (a,f) control (untreated cells). (b,g) 5-ALA-TPP-CTAC©AuNTs. (c,h) 5-ALA-TPP-PSS@CTAC©AuNTs. (d,i) IR-5-ALA-TPP-CTAC©AuNTs. (e,j) IR-5-ALA-TPP-PSS@CTAC©AuNTs. PI stains whose cells' DNA membrane is lost with red emission. In control, cells show no red emission is noticed, inferring no cell death. IR refers to irradiation. Scale bar – 125 µm/ 20× magnification).

**
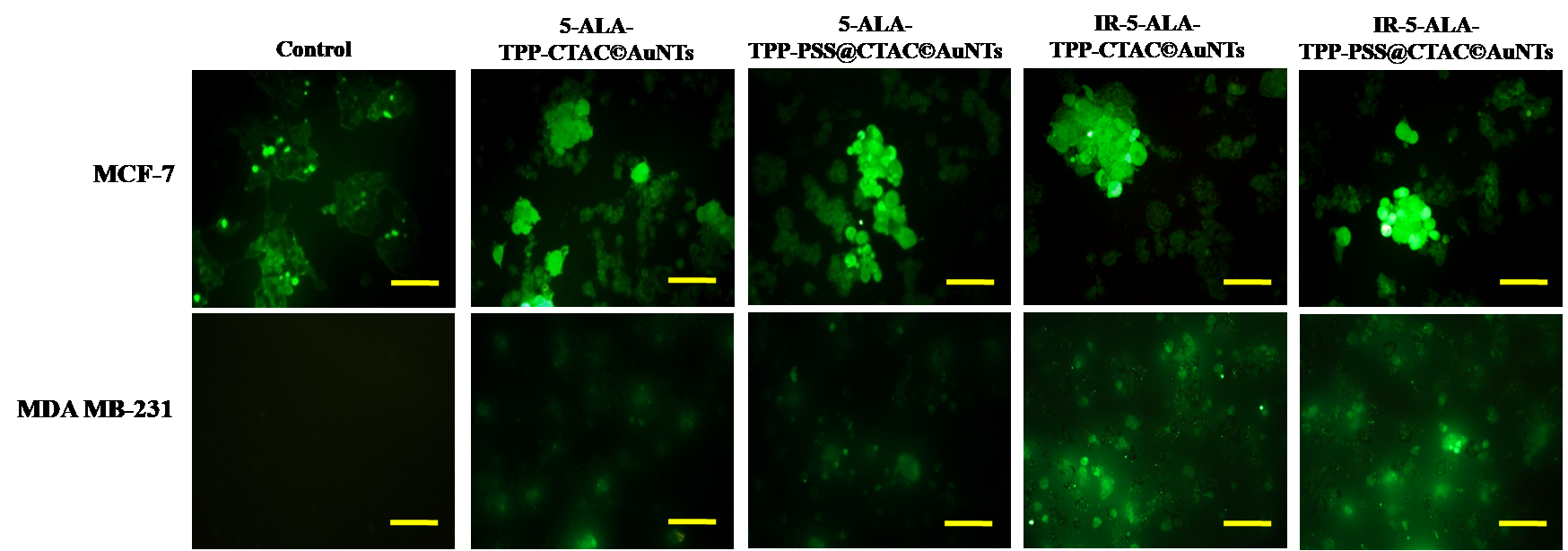
**

**SI, Figure 5.** Generation of reactive oxygen species (DCFH-DA) on breast cancer cells (MCF-7 and MDA-MB-231) after treatment with gold nanoconjugates with and without irradiation in the presence of 5-ALA. (a,f) control (untreated cells). (b,g) 5-ALA-TPP-CTAC©AuNTs. (c,h) 5-ALA-TPP-PSS@CTAC©AuNTs. (d,i) IR-5-ALA-TPP-CTAC©AuNTs. (e,j) IR-5-ALA-TPP-PSS@CTAC©AuNTs. DCFH-DA stains cells in green upon generation of ROS. No green emission is noticed. IR refers to irradiation. Scale bar – 125 µm/ 20× magnification).

**
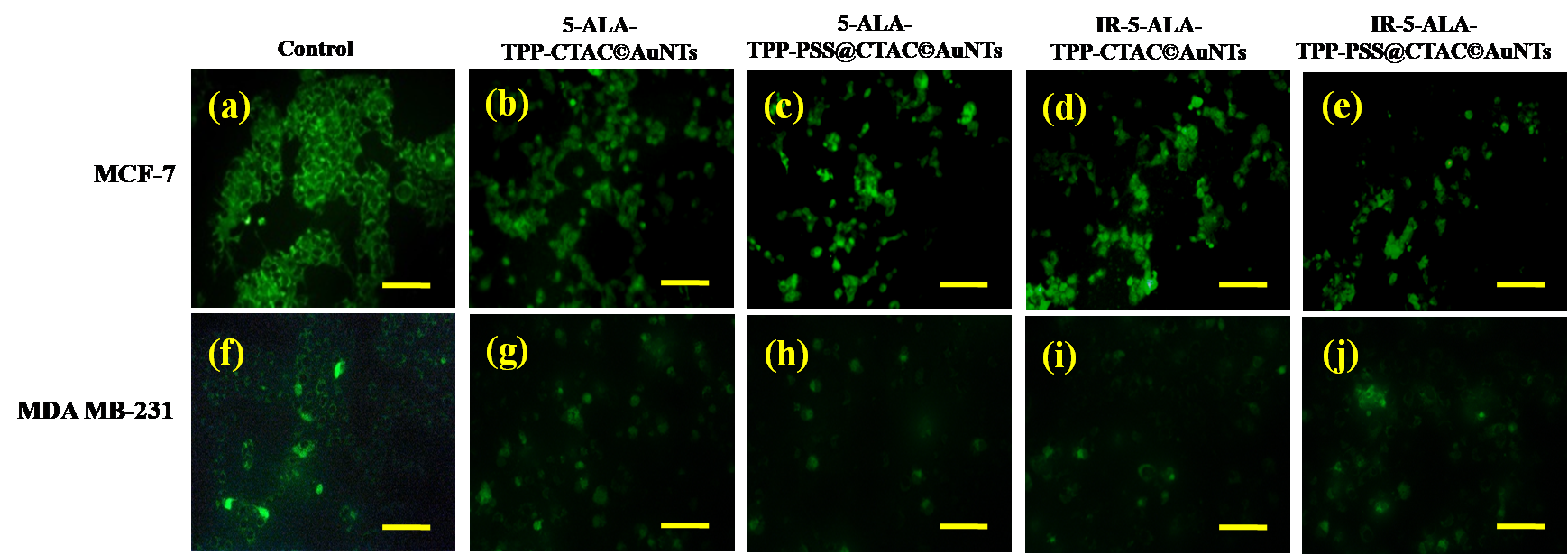
**

**SI, Figure 6.** Mitochondrial membrane permeation (ΔΨm, rhodamine-123) on breast cancer cells (MCF-7 and MDA-MB-231) after treatment with gold nanoconjugates with and without irradiation in the presence of 5-ALA. (a,f) control (untreated cells). (b,g) 5-ALA-TPP-CTAC©AuNTs. (c,h) 5-ALA-TPP-PSS@CTAC©AuNTs. (d,i) IR-5-ALA-TPP-CTAC©AuNTs. (e,j) IR-5-ALA-TPP-PSS@CTAC©AuNTs. An increase in green emissions infers healthy mitochondria. Decreased green emission confirms mitochondrial membrane permeation due to dysregulation in mitochondria. IR refers to irradiation. Scale bar – 125 µm/ 20× magnification).

**
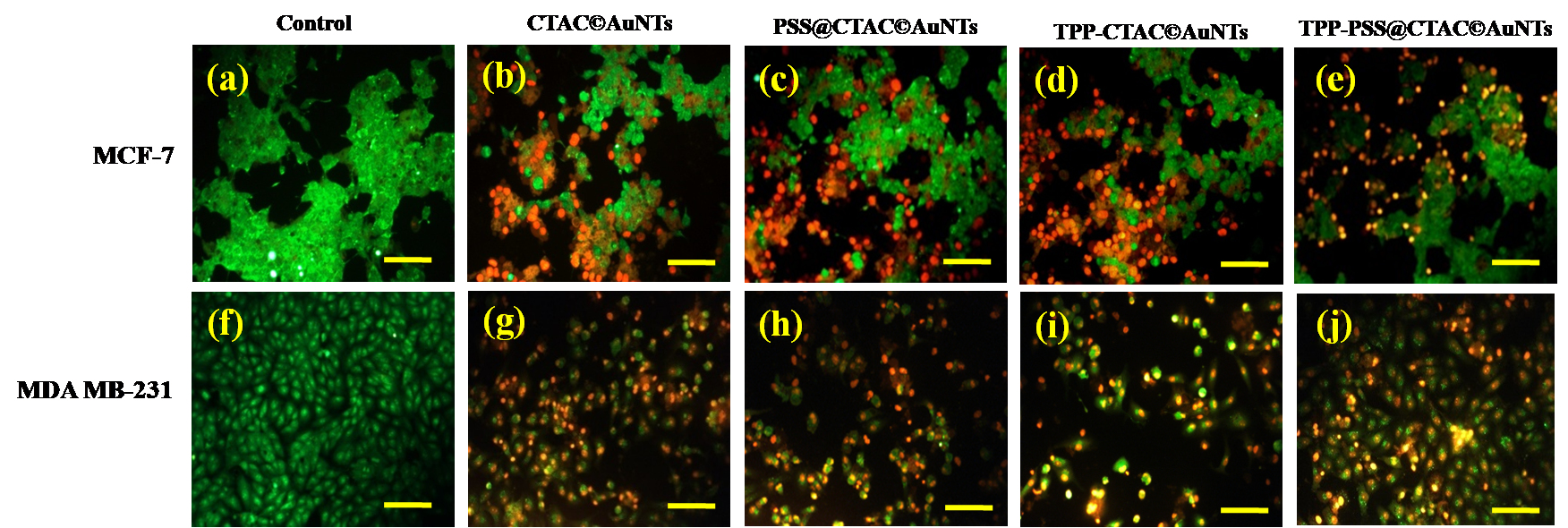
**

**SI, Figure 7.** Dual staining (AO/EtBr) on breast cancer cells (MCF-7 and MDA-MB-231) after treatment with AuNTs with and without TPP. (a,f) control (untreated cells). (b,g) CTAC©AuNTs. (c,h) PSS@CTAC©AuNTs. (d,i) TPP-CTAC©AuNTs. (e,j) TPP-PSS@CTAC©AuNTs. AO stains both live and dead cells in green, and EtBr stains DNA that undergo apoptosis. IR refers to irradiation. Scale bar – 125 µm/ 20× magnification).


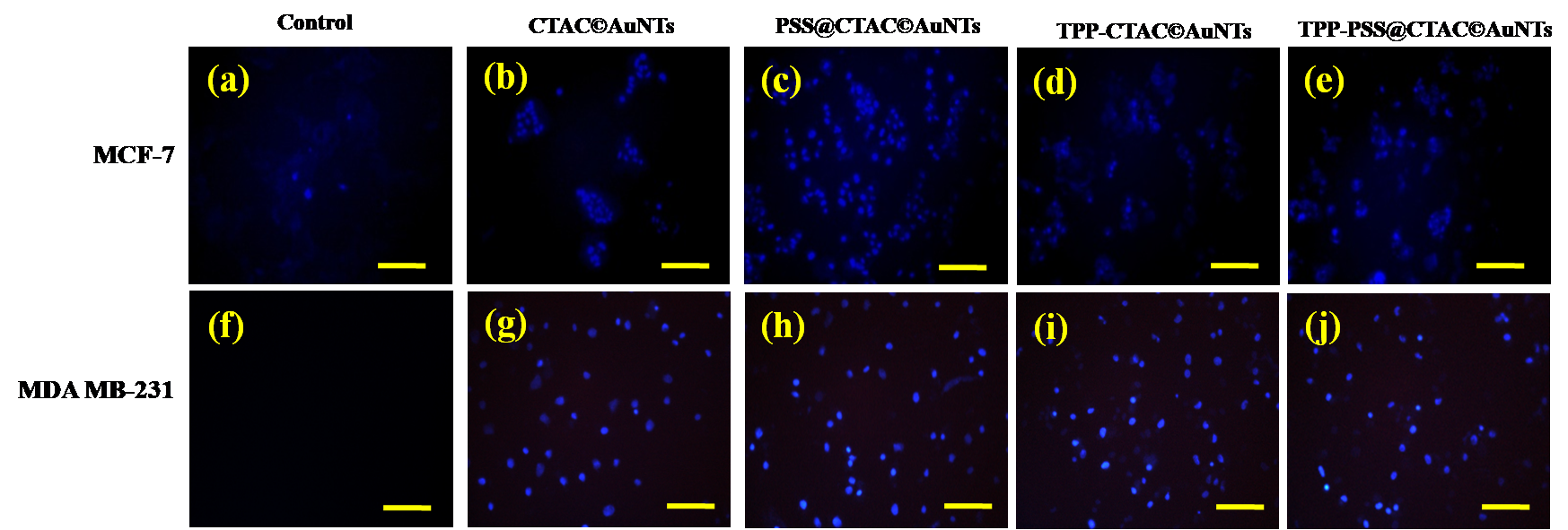


**SI, Figure 8.** Nuclear staining (Hoechst 33344) on breast cancer cells (MCF-7 and MDA-MB-231) after treatment with AuNTs with and without TPP. (a,f) control (untreated cells). (b,g) CTAC©AuNTs. (c,h) PSS@CTAC©AuNTs. (d,i) TPP-CTAC©AuNTs. (e,j) TPP-PSS@CTAC©AuNTs. Hoechst 33344 stains AT-rich regions of DNA whose cell membrane is damaged. In control, cells show no blue emission is noticed, inferring no cell death. IR refers to irradiation. Scale bar – 125 µm/ 20× magnification).


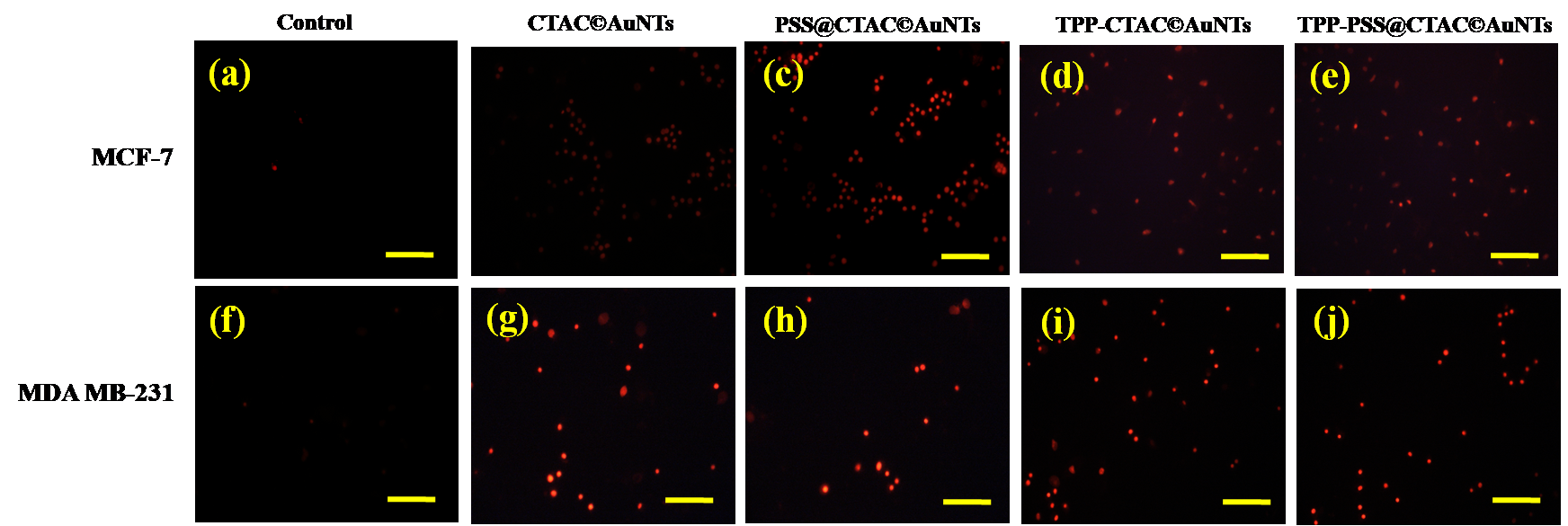


**SI, Figure 9.** DNA staining (PI) on breast cancer cells (MCF-7 and MDA-MB-231) after treatment with gold nanoconjugates with AuNTs with and without TPP. (a,f) control (untreated cells). (b,g) CTAC©AuNTs. (c,h) PSS@CTAC©AuNTs. (d,i) TPP-CTAC©AuNTs. (e,j) TPP-PSS@CTAC©AuNTs. PI stains whose cells' DNA membrane is lost with red emission. In control, cells show no red emission is noticed, inferring no cell death. IR refers to irradiation. Scale bar – 125 µm/ 20× magnification).


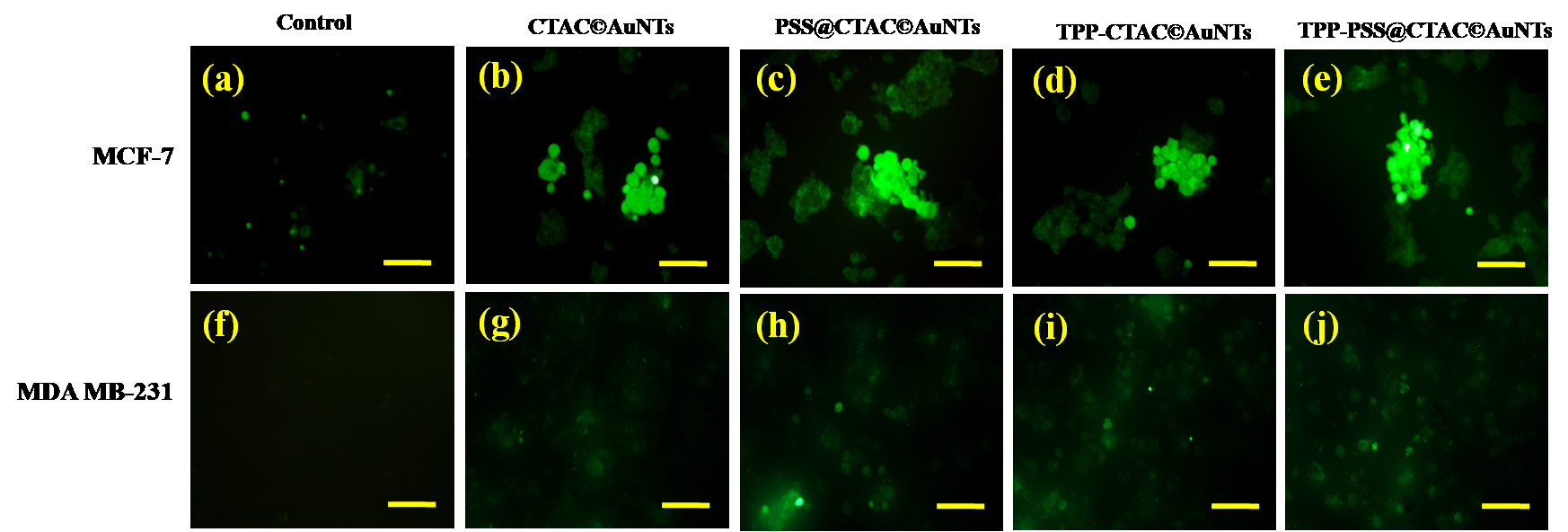


**SI, Figure 10.** Reactive oxygen species (DCFH-DA) on breast cancer cells (MCF-7 and MDA-MB-231) after treatment with AuNTs with and without TPP. (a,f) control (untreated cells). (b,g) CTAC©AuNTs. (c,h) PSS@CTAC©AuNTs. (d,i) TPP-CTAC©AuNTs. (e,j) TPP-PSS@CTAC©AuNTs. DCFH-DA stains cells in green upon generation of ROS. No green emission is noticed. IR refers to irradiation. Scale bar – 125 µm/ 20× magnification).

**
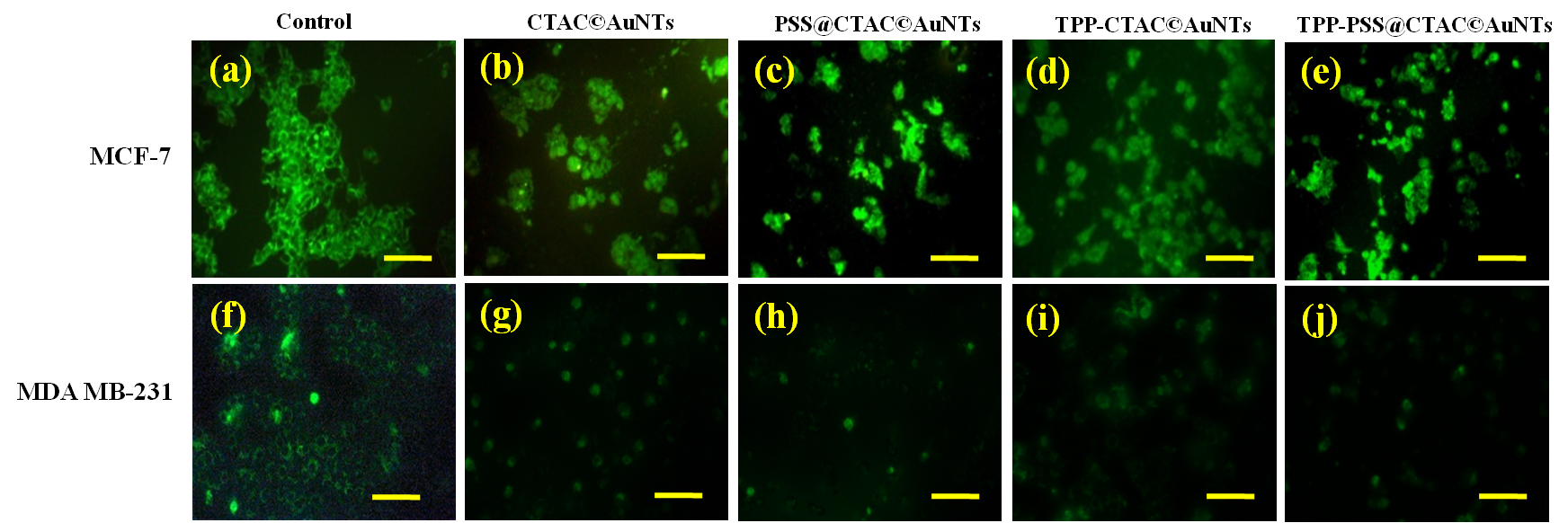
**

**SI, Figure 11.**Mitochondrial membrane permeation (ΔΨm, rhodamine-123) on breast cancer cells (MCF-7 and MDA-MB-231) after treatment with AuNTs with and without TPP. (a,f) control (untreated cells). (b,g) CTAC©AuNTs. (c,h) PSS@CTAC©AuNTs. (d,i) TPP-CTAC©AuNTs. (e,j) TPP-PSS@CTAC©AuNTs. An increase in green emissions infers healthy mitochondria. Decreased green emission confirms mitochondrial membrane permeation due to dysregulation in mitochondria. IR refers to irradiation. Scale bar – 125 µm/ 20× magnification).


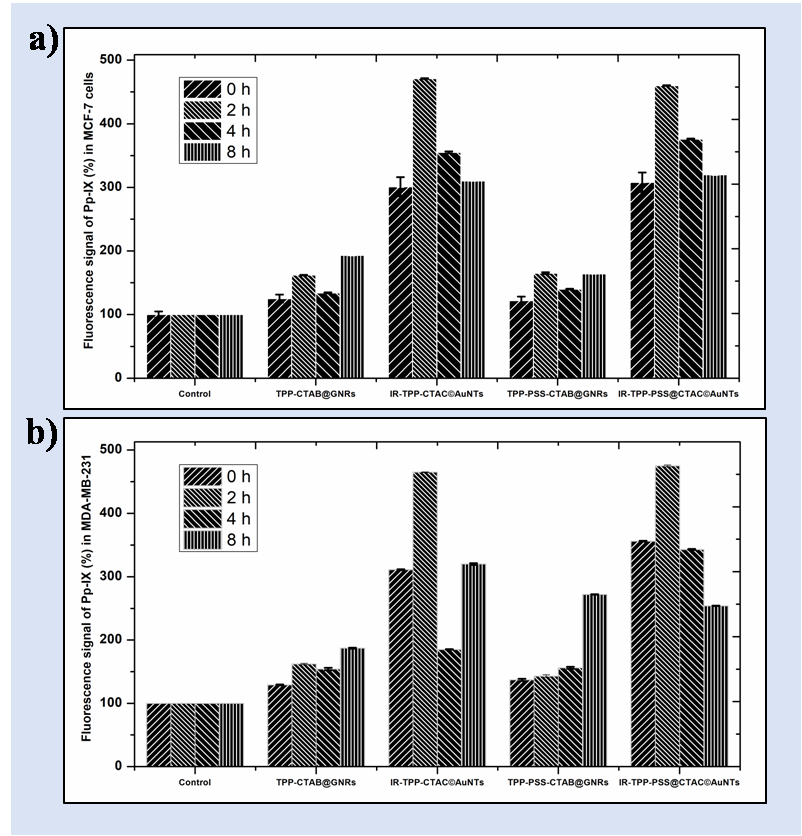


**SI, Figure 12.** Mean fluorescence Intensity of 5-ALA (0.5 mM) induced PDT in the presence of gold nanoconjugates at different time intervals (0 – 8 h).

**
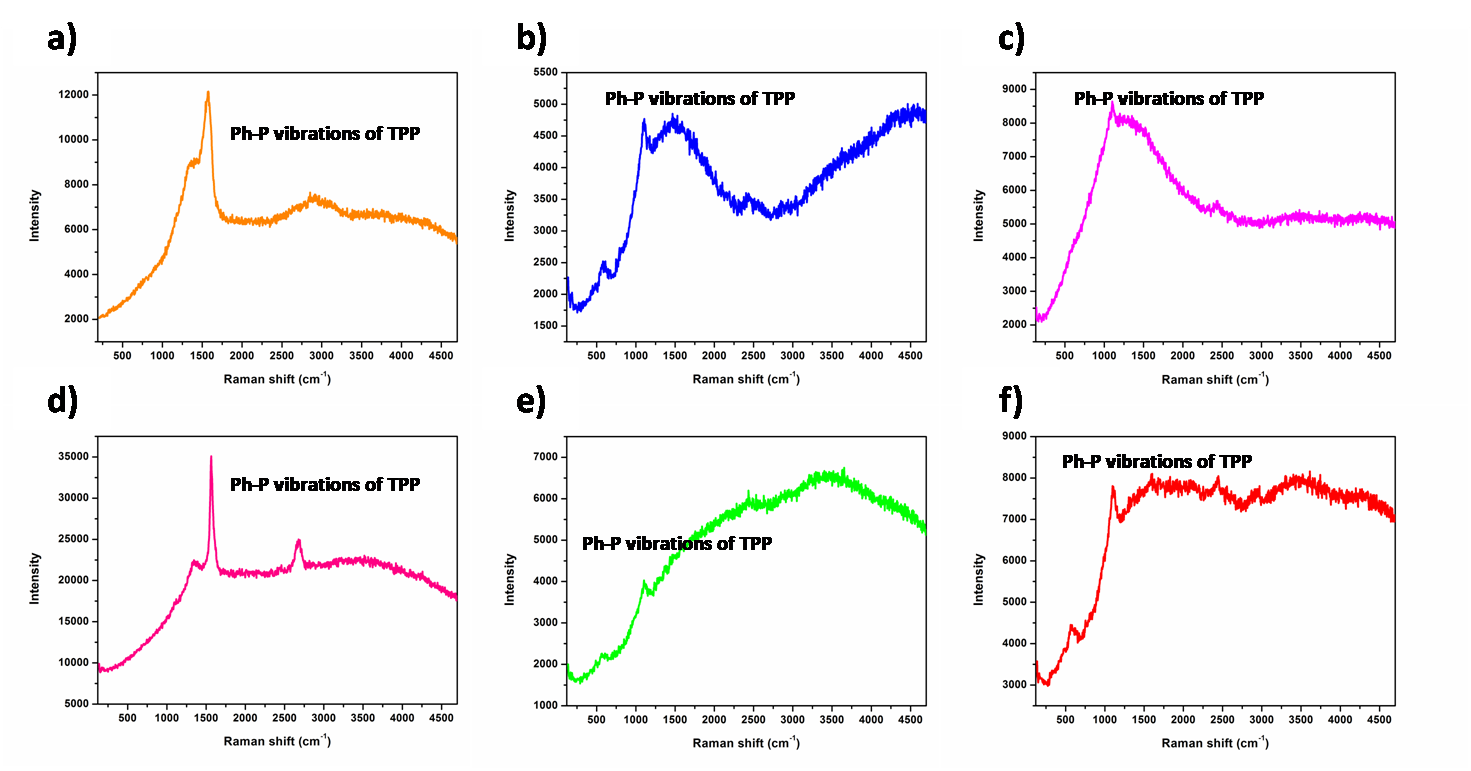
**

**SI, Figure 13.** Raman spectra of TPP co-localized with in MCF-7 (a-c) and MDA-MB-231 (d-f) breast cancer cell lines. (a, d) TPP. (b,e) TPP-CTAC©AuNTs. TPP-PSS@CTAC©AuNTs.


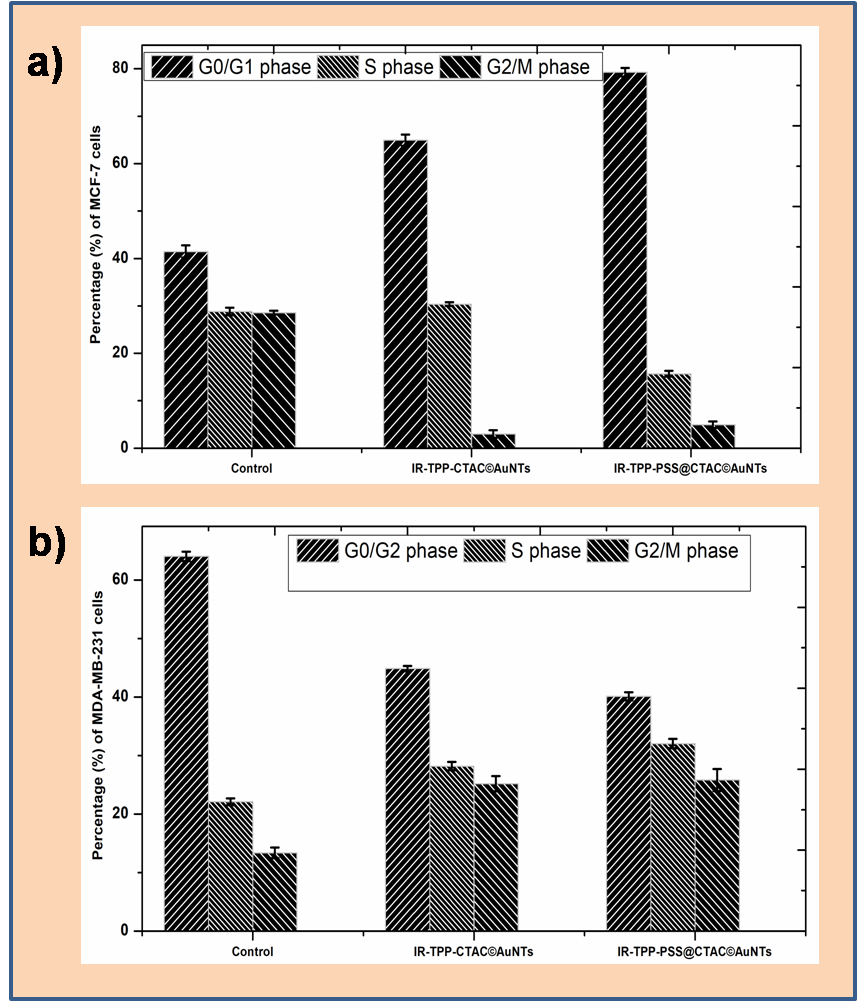


**SI, Figure 14.** The bar chart represents the percentage of cell cycle arrest upon treatment with gold nanoconjugates in the presence of 5-ALA mediated PDT on the breast cancer cells.

**
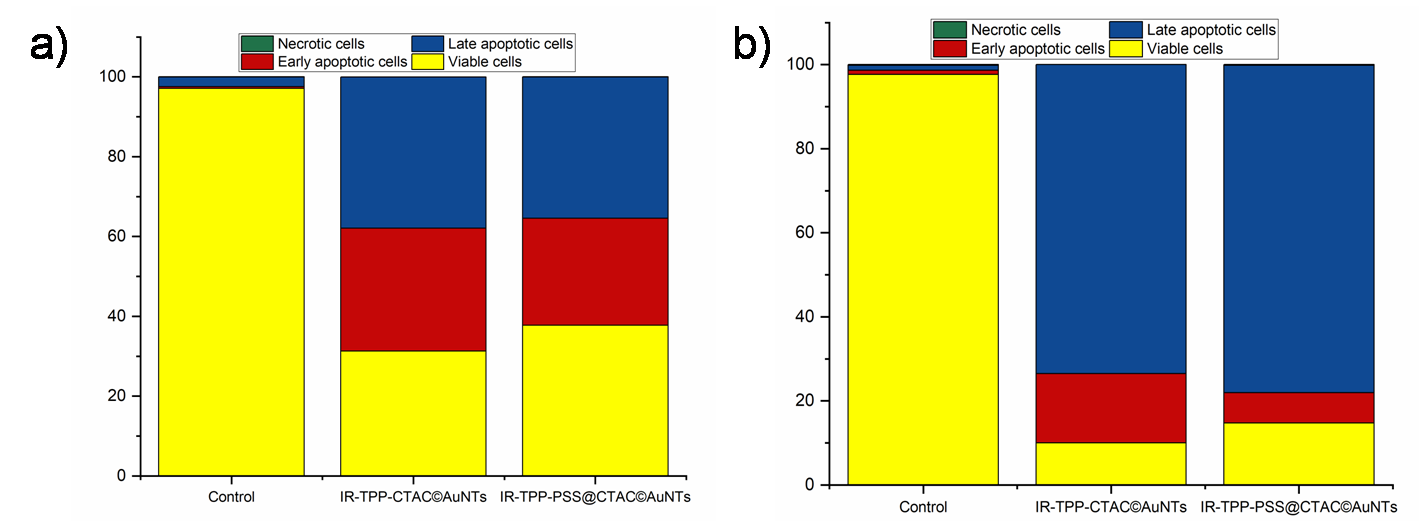
**

.

**SI, Figure 15.** Percentage of viable and apoptotic MCF-7 and MDA-MB-231 cells upon treatment with gold nanoconjugates in the presence of 5-ALA mediated PDT.


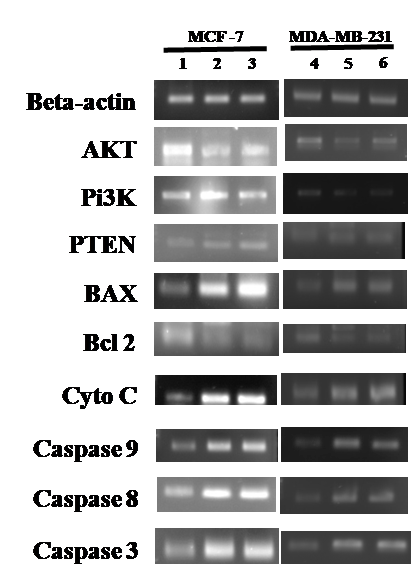


**SI, Figure 16.** mRNA expression studies of pro and anti-apoptotic genes upon 5-ALA-based PDT treatment. (1) Control (untreated MCF-7 cells). (2) IR-5-ALA-TPP-CTAC©AuNTs (IC_50_ – 0.71 ± 0.14 *µ*g/mL). (3) IR-5-ALA-TPP-PSS@CTAC©AuNTs (IC_50_ - 0.67 ± 0.89 *µ*g/mL). (4) Control (untreated MDA-MB-231 cells). (5) IR-5-ALA-TPP-CTAC©AuNTs (IC_50_ - 0.78 ± 0.55 *µ*g/mL). (6) IR-5-ALA-TPP-PSS@CTAC©AuNTs (IC_50_ - 0.58 ± 0.23 *µ*g/mL).


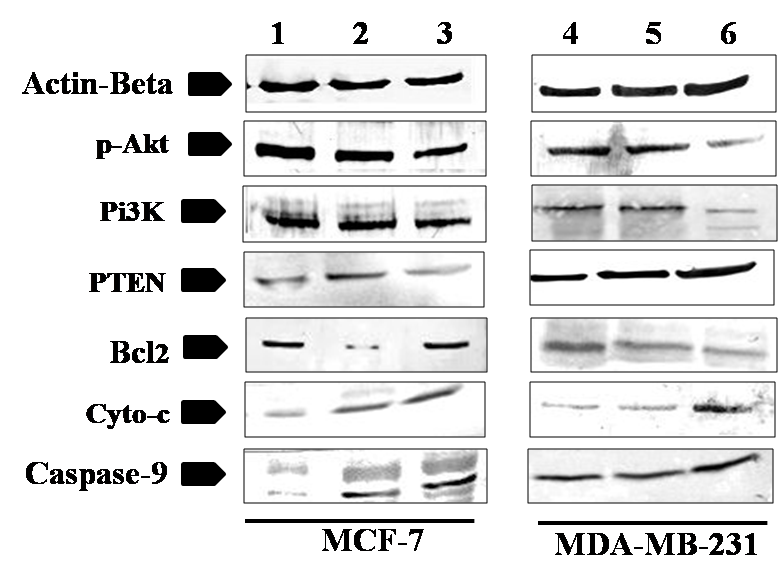


**SI, Figure 17.** Protein expression studies of pro and anti-apoptotic proteins upon 5-ALA-based PDT treatment. (1) Control (untreated MCF-7 cells). (2) IR-5-ALA-TPP-CTAC©AuNTs (IC_50_ – 0.71 ± 0.14 *µ*g/mL). (3) IR-5-ALA-TPP-PSS@CTAC©AuNTs (IC_50_ - 0.67 ± 0.89 *µ*g/mL). (4) Control (untreated MDA-MB-231 cells). (5) IR-5-ALA-TPP-CTAC©AuNTs (IC_50_ - 0.78 ± 0.55 *µ*g/mL). (6) IR-5-ALA-TPP-PSS@CTAC©AuNTs (IC_50_ - 0.58 ± 0.23 *µ*g/mL).

**Unprocessed gels – Semi-Quantitative RT-PCR analysis**


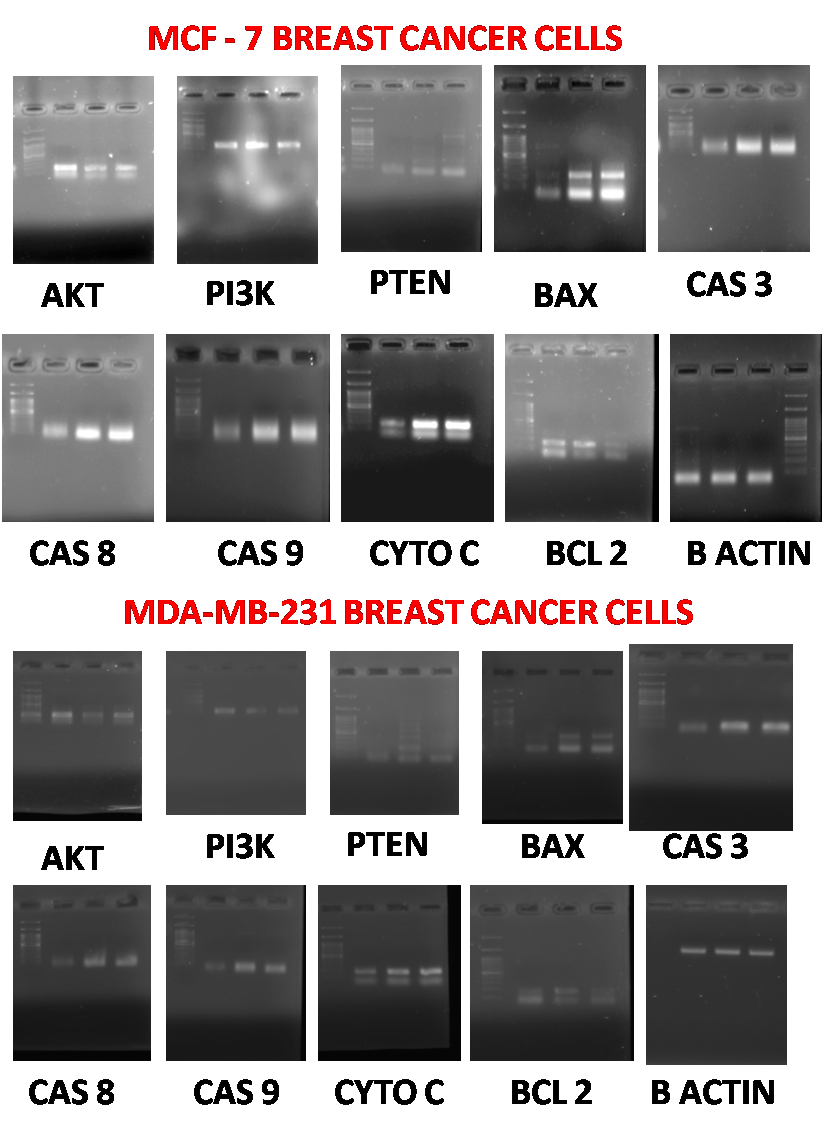


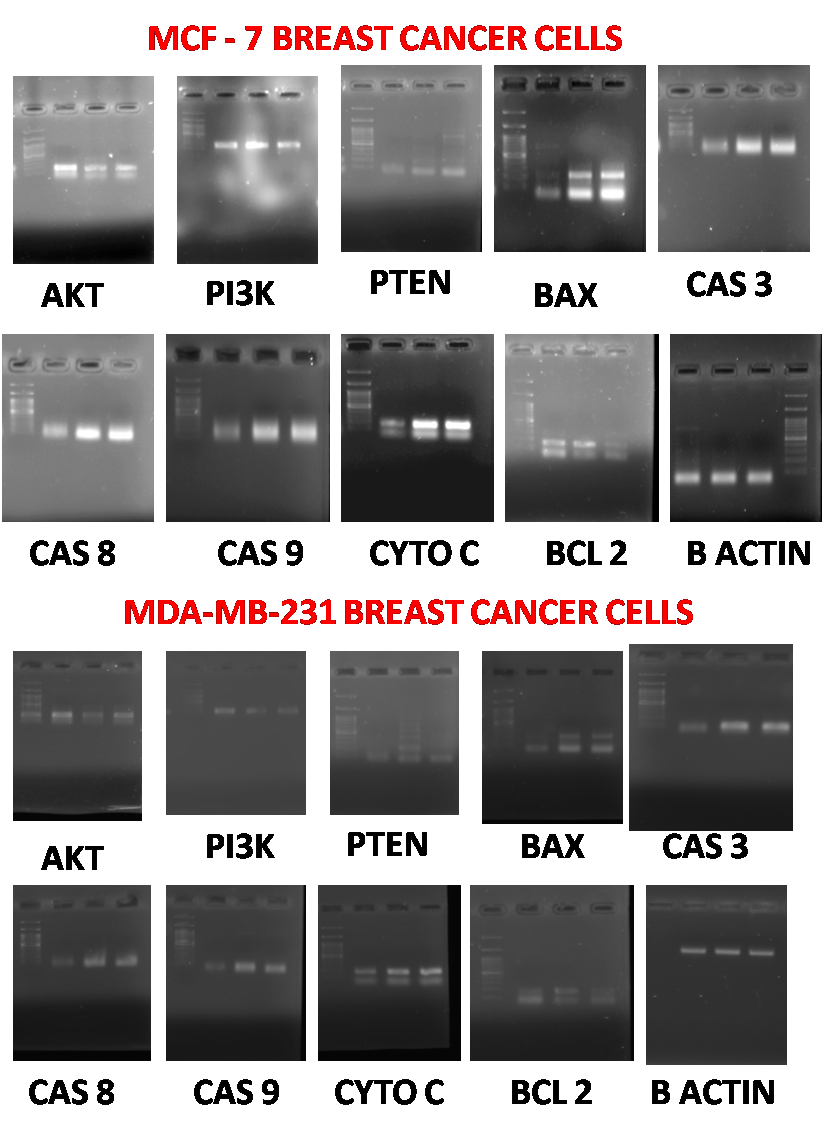


**Unprocessed blots – Western blot analysis**


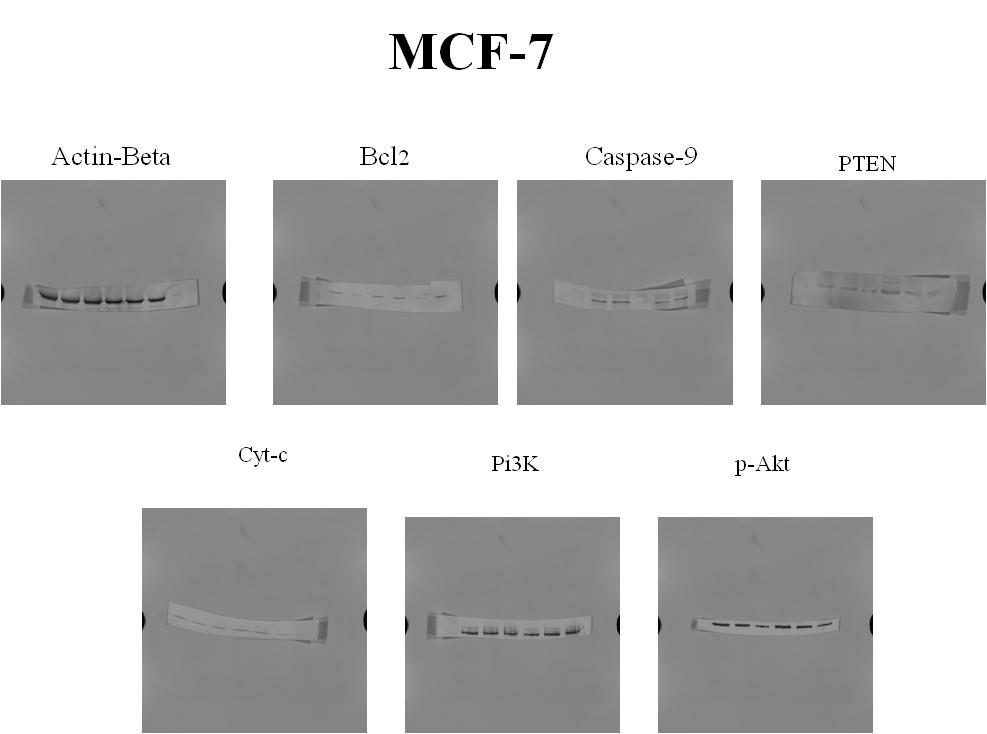


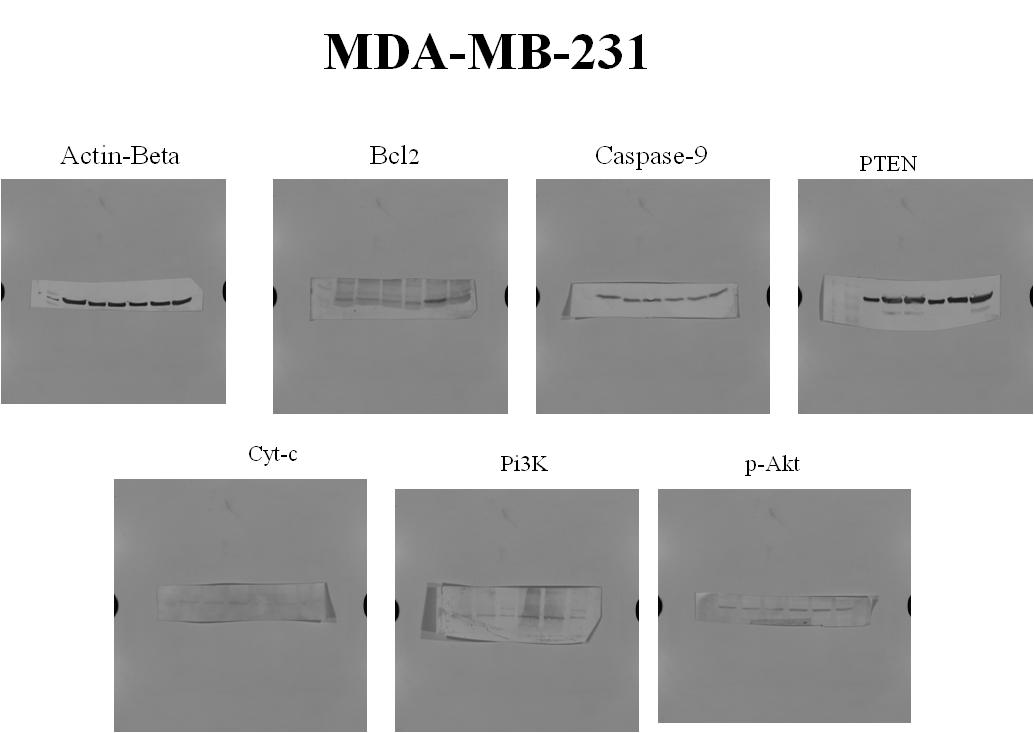

Supplement: Supplementary file 1 — Supplementary Information. [file 41598_2023_28678_MOESM1_ESM.doc]
